# An unprecedented insight into the catalytic mechanism of copper nitrite reductase from atomic-resolution and damage-free structures

**DOI:** 10.1126/sciadv.abd8523

**Published:** 2021-01-01

**Authors:** Samuel L. Rose, Svetlana V. Antonyuk, Daisuke Sasaki, Keitaro Yamashita, Kunio Hirata, Go Ueno, Hideo Ago, Robert R. Eady, Takehiko Tosha, Masaki Yamamoto, S. Samar Hasnain

**Affiliations:** 1 Molecular Biophysics Group, Life Sciences Building and Institute of Systems, Molecular and Integrative Biology, Faculty of Health and Life Sciences, University of Liverpool, Liverpool L69 7ZB, UK.; 2 Department of Biological Sciences, Graduate School of Science, The University of Tokyo, 7-3-1 Hongo, Bunkyo-ku, Tokyo 113-0033, Japan.; 3 RIKEN SPring-8 Center, 1-1-1 Kouto, Sayo, Hyogo 679-5148, Japan.

## Abstract

Copper-containing nitrite reductases (CuNiRs), encoded by *nirK* gene, are found in all kingdoms of life with only 5% of CuNiR denitrifiers having two or more copies of *nirK*. Recently, we have identified two copies of *nirK* genes in several α-proteobacteria of the order Rhizobiales including *Bradyrhizobium* sp. ORS 375, encoding a four-domain heme-CuNiR and the usual two-domain CuNiR (*Br*^2D^NiR). Compared with two of the best-studied two-domain CuNiRs represented by the blue (*Ax*NiR) and green (*Ac*NiR) subclasses, *Br*^2D^NiR, a blue CuNiR, shows a substantially lower catalytic efficiency despite a sequence identity of ~70%. Advanced synchrotron radiation and x-ray free-electron laser are used to obtain the most accurate (atomic resolution with unrestrained SHELX refinement) and damage-free (free from radiation-induced chemistry) structures, in as-isolated, substrate-bound, and product-bound states. This combination has shed light on the protonation states of essential catalytic residues, additional reaction intermediates, and how catalytic efficiency is modulated.

## Teaser

Highly accurate SR structures and damage-free XFEL structures provide detailed insight into catalytic mechanism of CuNiR.

## INTRODUCTION

Understanding catalysis by enzymes underpinned by high-resolution three-dimensional structures has attracted major attention from structural enzymologists who have catalyzed the advances in experimental capabilities by continuing to push the capabilities of synchrotron radiation (SR) crystallography improving brilliance of x-ray beams and detectors and have recently opened the possibility of obtaining damage-free, free from radiation-induced chemistry (FRIC), structures. We have applied these advanced methods to obtain the most accurate [SR structures at resolutions of ~1 Å enabling unrestrained, SHELX refinement for any copper-containing nitrite reductase (CuNiR)] and damage-free x-ray free-electron laser (XFEL)–FRIC structures, in the as-isolated, substrate-bound, and product-bound states, again to high resolutions of 1.3 Å. For all of these structures, optical spectra were collected on single crystals. This combination has led to a real scientific advance in the understanding of the catalytic mechanism of NiRs, which are part of the nitrogen cycle.

The global nitrogen cycle has seen a marked imbalance over the years as a result of anthropogenic factors, primarily from an increased use of artificial nitrogenous fertilizers and the burning of fossil fuels (*1*, *2*). Major contributors to the nitrogen cycle are several α-proteobacteria of the order Rhizobiales, collectively Rhizobia, which uniquely form specific symbiotic relationships with leguminous plants such as soybean (*3*). In addition to nitrogen fixation, Rhizobia are also able to use fixed nitrogen, in the form of nitrate, as a replacement for dioxygen during respiratory adenosine 5′-triphosphate synthesis through a dissimilatory denitrification process (*4*, *5*). In doing so, they also contribute considerably to the removal of bioavailable fixed nitrogen from terrestrial sources and are responsible for a substantial balance in the world’s fixed nitrogen supply (*3*, *6*, *7*).

Denitrification involves the sequential reduction of nitrate (NO_3_^−^) and/or nitrite (NO_2_^−^) to dinitrogen (N_2_) through gaseous intermediates nitric oxide (NO) and nitrous oxide (N_2_O) in a four-step process involving specific reductases: NO_3_^−^ → NO_2_^−^ → NO → N_2_O → N_2_ (*4*, *5*). A major by-product of denitrification is the greenhouse gas N_2_O, the largest and more potent ozone-depleting substance than CO_2_ (*8*, *9*). In addition to their contribution to nitrogen fixation in worldwide agriculture, Rhizobia also make a substantial contribution to total denitrification and, consequently, atmospheric N_2_O production and hence are of major importance to both environment and agriculture (*7*, *10*).

CuNiRs, encoded by the *nirK* gene, are key enzymes in the denitrification pathway, as it is at this point that the bioavailable fixed nitrogen gets recycled back to the atmosphere. These redox-active metalloenzymes are responsible for catalyzing the one-electron and two-proton reduction of NO_2_^−^ to gaseous product NO in the first committed step of this pathway (NO_2_^−^ + 2H^+^ + *e*^−^→NO + H_2_O) (*4*). CuNiRs are a highly conserved enzyme family that use cupredoxin-like domains to assemble a catalytic core made up of type 1 Cu (T1Cu) and type 2 Cu (T2Cu) (*11*). Structures of the well-characterized homotrimeric two-domain CuNiRs show the catalytic T2Cu center located at the interface of two monomers and is linked to the electron-donating T1Cu center by a 12 Å Cys-His bridge. Two invariant active-site pocket residues Asp_CAT_ and His_CAT_ are involved in substrate binding and provision of protons during catalysis, and a conserved Ile_CAT_ residue provides steric control of ligand binding to the T2Cu (*12*–*14*). Depending on the organism, the oxidized enzymes are blue or green, arising from differences in the geometry of the copper ligation of the T1Cu, often accompanied by a different physiological donor. These enzymes are located in the periplasm, and each class receives an electron from different partner proteins either cytochrome or a cupredoxin (*15*). To date, structural studies at atomic resolution of as-isolated, substrate-bound, and product-bound species are restricted to the green CuNiRs from *Achromobacter cycloclastes* (*Ac*NiR) and *Alcaligenes faecalis* (*Af*NiR) (*16*–*18*). In contrast, ligand-bound species of blue NiRs such as *Achromobacter xylosoxidans* (*Ax*NiR) have proved intractable to even high-resolution studies and are known to very limited resolutions of 2.34 and 2.8 Å (*19*, *20*).

We have recently identified a blue CuNiR from *Bradyrhizobium* ORS 375 of the order Rhizobiales (*Br*^2D^NiR) (*21*). The catalytic efficiency of *Br*^2D^NiR was found to be substantially lower than two of the well-studied two-domain CuNiRs representing the blue (*Ax*NiR) and green (*Ac*NiR) subclasses, despite a sequence identity of ~70% (*21*). The blue crystals of *Br*^2D^NiR proved amenable to soaking of substrate and yielded highly diffracting crystals, providing a unique opportunity to obtain atomic-resolution structures of up to 1 Å with synchrotron x-rays and damage-free FRIC structures using XFEL, to ~1.3 Å resolution of as-isolated, nitrite- and nitric oxide–bound states. The significantly higher resolution of synchrotron structures allowed unrestrained SHELX refinement, while the XFEL structures of as-isolated and ligand-bound states allowed us to define these catalytically critical states without any x-ray–induced damage and unintended X-ray induced redox chemistry. We have generated the product in situ by using substrate-soaked crystals with full occupancy of substrate at the catalytic T2Cu site and treating them with a chemical reductant to initiate the turnover. This has allowed characterization of the stable nitrosyl intermediate with “side-on” binding unequivocally refuting the hypothesis that “the proton-coupled electron transfer to the nitrite-bound T2Cu leads to reduction of nitrite to form NO, which is released without forming a Cu-nitrosyl species” (*22*–*24*). Together, these results highlight structural aspects that are important for defining and regulating catalysis in CuNiR and thus should be considered before designing synthetic chemical systems that are capable of performing this widespread biological function in a variety of environmental and biomedical applications (*25*–*27*).

## RESULTS

### Structure of *Br*^2D^NiR at 1.1 Å atomic resolution using synchrotron-radiation crystallography

**Fig. 1:**
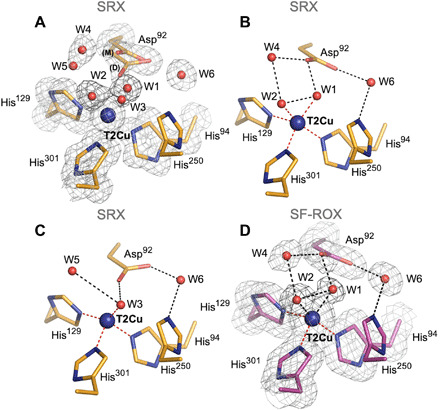
T2Cu site in as-isolated *Br*^2D^NiR: SRX v SF-ROX structures. (**A**) In the 1.1 Å resolution SRX structure (orange), five waters (W1 to W5) and two conformations of Asp^92^: proximal (main and distorted) are clearly observed. (**B**) In one active-site conformation, positions of W1 and W2 at the T2Cu site are correlated with the main proximal conformation of Asp^92^. (**C**) In alternative conformation, water W3 is correlated with the distorted conformation of Asp^92^. (**D**) T2Cu site in the 1.3 Å XFEL FRIC structure (magenta) clearly shows two full-occupancy waters (W1 and W2) and Asp^92^ in main proximal conformation only. 2*F*_o_ − *F*_c_ electron density map, contoured at 1σ level, colored in gray. Water molecules are shown as red spheres, and T2Cu is shown as a blue sphere. Distances for possible bonding are shown as black dashed lines.

To characterize this new two-domain CuNiR enzyme, we obtained a 1.1 Å atomic-resolution structure in its as-isolated state using conventional synchrotron-radiation crystallography (SRX) at 100 K. To provide rigor and reduce the ambiguities in the assignments of multiple conformations of active-site ligands and define SDs of individual bond distances and angles of residues in the catalytic core, we used SHELX-97 (tables S2 to S4) [estimated standard deviation (e.s.d.) values available from SHELX are given in brackets] (*28*). A notable observation was the presence of two waters: (W1) and (W2) concurrently bound to the catalytic T2Cu site in one conformation (Fig. 1, A and B) and an alternative water (W3) in second conformation (Fig. 1, A and C). Two waters, W1 and W2, with equal occupancies (0.67) are 2.49 Å apart and bound to the T2Cu atom at distances of 1.940(3) Å and 2.162(3) Å, respectively (table S3). The occupancies of ~0.7 for these two waters strongly argue for their coexistence rather than alternate conformations of the same water. An alternative third water, W3, bound at 2.018(3) Å with ideal tetrahedral geometry to T2Cu with an occupancy of 0.33 represents the site in molecules where both W1 and W2 are absent (Fig. 1, A and C). The catalytically important Asp_CAT_ residue (Asp^92^) is present in both the main (occupancy, 0.67) and distorted (occupancy, 0.33) conformations of its proximal position, as first seen in XFEL structures of *Ac*NiR (*29*), with these facing toward the T2Cu site (Fig. 1). Analysis of the carboxyl group bond lengths (O^δ1^─C^γ^ and O^δ2^─C^γ^) in these conformations with the ideal bond length (fig. S1, D and E, and table S4) suggests the possibility that proximal position is negatively charged [1.25 Å (3)] and [1.26 Å (3)], while the carboxyl group in distorted proximal position is protonated [1.25 Å (7)] and [1.29 Å (8)] and neutral. Although seen together in our atomic-resolution structure, these two Asp_CAT_ conformations do not occur simultaneously but are only present either alongside W1 and W2 (main conformation) (Fig. 1B) or alongside W3 (distorted conformation) (Fig. 1C). In its main conformation, the carboxyl oxygen (O^δ2^) atom of Asp_CAT_ is hydrogen bonded with water W1 and a substrate channel water (W4) with 0.5 occupancy at distances 2.52 and 2.45 Å, respectively, while O^δ2^ atom of the distorted conformation is bound to W3 (3.13 Å). W4 can also hydrogen bond with W2, while a second position at 0.5 occupancy (W5) can hydrogen bond with W3 only at same distances (2.98 Å). All hydrogen bond contact distances are listed in table S5.

**Fig. 2:**
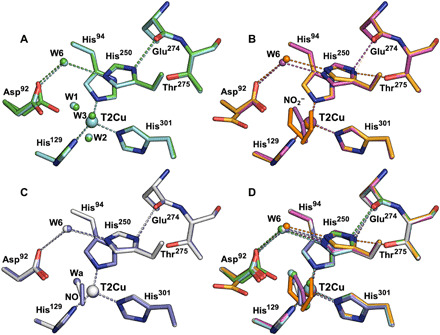
Rotation of His^250^ (His_CAT_) imidazole in each structure. (**A**) N^δ1^ atom of His_CAT_ in as-isolated *Br*^2D^NiR SRX (green) v SF-ROX (cyan) structures, both forming a strong hydrogen bond with main-chain O atom of Glu^274^. (**B**) His_CAT_ in nitrite-bound *Br*^2D^NiR SRX (orange) v SF-ROX (magenta) structures; N^δ1^ atom of His_CAT_ forms a strong hydrogen bond with main-chain O atom of Glu^274^ in nitrite-bound SF-ROX structure but is rotated in nitrite-bound SRX structure to form a new hydrogen bond with O^γ1^ of Thr^275^. (**C**) N^δ1^ atom of His_CAT_ in NO-bound/T1Cu-reduced *Br*^2D^NiR SRX (white) v SF-ROX (purple) structures forms a strong hydrogen bond with main-chain O atom of Glu^274^. (**D**) Superimposition of all *Br*^2D^NiR structures, His_CAT_ rotation and change in hydrogen bond position, is only observed in atomic-resolution SRX nitrite-bound structure.

The catalytically important His_CAT_ residue (His^250^) has its imidazole ring rotated toward Glu^274^ forming a strong hydrogen bond by its N^δ1^ atom with the main-chain carbonyl oxygen of Glu^274^ at 2.67 Å. While its N^δ1^ atom is connected to Asp_CAT_ via water W6 (Figs. 1 and 2A). C─N─C angles associated with N^δ1^ and N^ε2^ atoms of His_CAT_ are 112.5°(1.6) and 106.4°(1.5), respectively, which corresponds to protonated N^δ1^ and nonprotonated N^ε2^ (fig. S1, A and B, and table S4) (*30*, *31*) when compared with imidazole groups in the Cambridge Structural Database (*32*).

### Damage-free XFEL FRIC structure of *Br*^2D^NiR at 1.3 Å resolution

**Fig. 3:**
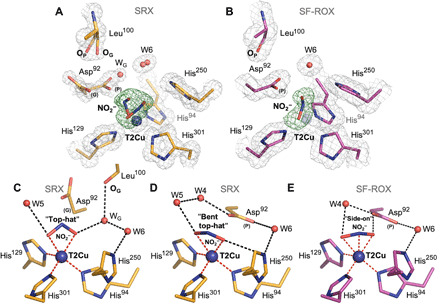
T2Cu site in nitrite-bound *Br*^2D^NiR: SRX v SF-ROX structures. (**A**) The 1 Å SRX structure (orange) showing two top-hat conformations of nitrite and Asp^92^ (proximal and gatekeeper). A partial water (W_G_) and movement of Leu^100^ oxygen atom (O_P_ > O_G_) are also seen. (**B**) The 1.3 Å XFEL FRIC structure (magenta) showing single nitrite in side-on position and Asp^92^ in proximal conformation. (**C**) SRX T2Cu site with bidentate-bonded top-hat nitrite present. (**D**) Alternative T2Cu of SRX structure with “bent top-hat” conformation of nitrite. (**E**) SF-ROX structure with horizontal side-on coordination of nitrite. Bonding and coordination of waters and atoms are shown for all. OMIT *F*_o_ − *F*_c_ electron density maps around nitrite molecules are contoured at 5σ level and colored green. 2*F*_o_ − *F*_c_ electron density maps are contoured at 1σ level in gray. Water molecules are shown as red spheres, and T2Cu is shown as a blue sphere. Hydrogen bonds are shown as black dashed lines, and coordination distances to T2Cu are shown as red dashed lines.

To understand the nature of two waters seen bound to T2Cu in the atomic-resolution SRX structure, we obtained a 1.3 Å XFEL FRIC structure of *Br*^2D^NiR in its fully oxidized as-isolated state by using serial femtosecond rotational crystallography (SF-ROX) (*33*) technique at 100 K using XFEL. The oxidation state of the crystal was confirmed by ultraviolet-visible (UV/vis) microspectrophotometry before irradiation of one of the crystals at SACLA (SPring-8 Angstrom Compact free-electron LAser) (fig. S2). This damage-free FRIC structure obtained with pulses of SACLA of less than 10-fs duration (a time frame in which all of the atoms are frozen in space and time, as it is shorter than the rotational and vibrational frequencies) revealed the presence of two clear full-occupancy waters (W1) and (W2) bound to T2Cu, at distances of 2.05 and 1.94 Å, respectively, that are 2.51 Å apart. Asp_CAT_ is in main proximal conformation, and its O^δ2^ atom forms similar hydrogen bonds as in the SRX structure with full-occupancy waters W4 and W1 at distances of 2.81 and 2.39 Å, respectively (Fig. 1D). In a recent damage-free XFEL structure of *Af*NiR (*18*), a rotation of the His_CAT_ imidazole ring was reported when compared to its SRX structure. This rotation was suggested to be catalytically relevant, with His_CAT_ having the role of a redox-coupled proton switch, essential for CuNiR catalysis. However, our results differ with no change seen between conformation of His_CAT_ in the SRX and XFEL (FRIC) structures of as-isolated *Br*^2D^NiR (Fig. 3A). The imidazole ring of His_CAT_ residue in the XFEL’s FRIC structure is rotated toward Glu^274^ forming a hydrogen bond between His_CAT_ N^δ1^ atom and Glu^274^ O at 2.64 Å (Fig. 2A). We note that in the XFEL structure of *Af*NiR (*18*), a chloride present in the purification and crystallization stages had replaced the usual water ligand of the T2Cu. In the case of *Ax*NiR, SF-ROX structure had revealed dioxygen was ligated to T2Cu (*34*).

The electron-donating T1Cu site in both structures of *Br*^2D^NiR is similar to that of other two-domain CuNiRs, except the conformation of Met^136^, which is the terminal residue in an adjacent loop termed the “proton trigger loop” (Pro^132^-Met^136^). This loop connects to the proton-gated Cys-His electron transfer bridge. In *Br*^2D^NiR, Met^136^ is seen in only one conformation (flipped 180° away from His^140^) when compared to other two-domain CuNiRs, allowing a 2.79 Å hydrogen bond to form between T1Cu residue His^140^ and a new water (W10) as part of an ordered water network (fig. S3).

Two putative proton channels, one involving Asn^90^ and the other His^254^ (*Ax*NiR numbering), have been proposed with the Asn^90^ channel as the probable main proton channel, based on mutational and kinetic studies (*20*). We have examined these two proton channels in both our 1.1 Å SRX and 1.3 Å XFEL FRIC structures in relation to *Ax*NiR [Protein Data Bank (PDB): 10E1] and *Ac*NiR (PDB: 2BW4) atomic-resolution SRX structures and the 1.5 Å XFEL damage-free structure of *Ac*NiR (PDB: 6GSQ). The Asn^90^ channel in both SRX and XFEL structures of *Br*^2D^NiR shows no marked differences but is more similar to *Ac*NiR structures (fig. S4A) than *Ax*NiR structures (fig. S4B), with similar waters and hydrogen bonds constituting the network. Unusually, a half-occupancy water from the substrate channel (W7a) is favored in *Br*^2D^NiR Asn^90^ channel compared to *Ac*NiR and *Ax*NiR channels (W3/W2) to maintain strong bonding distances and efficient proton transfer (fig. S4, A and B). In *Br*^2D^NiR, Leu^107^ forms the entrance to the Asn^90^ channel in contrast to Asn^107^ (*Ax*NiR) and Gln^113^ (*Ac*NiR), which, similarly to green NiRs, results in a key hydrogen bond to Asn^90^ (as in blue *Ax*NiR) being abolished. Residue Gly^103^ substitutes this by allowing a hydrogen bond to be formed between its carbonyl oxygen atom and Asn^90^ as well as a water molecule within the channel (fig. S4C). The surface residue Ala^307^ forms the entrance of the substrate channel to the Asn^90^ channel, substituting a bulky phenylalanine residue present in other two-domain CuNiRs; this allows more water molecules into the channel compared to other CuNiRs. The His^254^ channel is conserved in all structures of *Br*^2D^NiR, *Ax*NiR, and *Ac*NiR. The overall preservation of both proton channels suggests that these channels provide alternative routes, allowing the enzyme to use either of them depending on the denitrifying conditions including pH and/or nitrite concentration.

### SRX structure of nitrite-bound enzyme at 1 Å atomic resolution

A 1 Å resolution structure of *Br*^2D^NiR with nitrite bound was obtained using one of the sophisticated SR crystallographic beamlines at the Diamond Light Source I03 equipped with DECTRIS PILATUS3 6M detector and crystal maintained at 100 K. The very high resolution was used to obtain the most accurate structural information by deploying SHELX. The OMIT (*F*_o_ − *F*_c_) electron density map clearly shows that a nitrite molecule binds to T2Cu in two different “top-hat” conformations (Fig. 3A). The oxygen atoms of the first top-hat orientated nitrite are coordinated to T2Cu at distances of 2.13(6) and 1.98(2) Å (Fig. 3C), while a “bent top-hat” nitrite is bound at slightly longer distances of 2.22(5) Å and 2.13(2) Å for O^1^ and O^2^ atoms, respectively (Fig. 3D). Unrestrained refinement with estimated standard deviations (e.s.d.’s) analysis also showed that geometries of the two nitrite molecules are slightly different, the bond lengths of the top-hat nitrite are longer [1.30(4) Å] with a bond angle of 119.6°(3.5), while in the bent top-hat nitrite, they are shorter and slightly asymmetric [O^1^─N 1.25(6)] and [O^2^─N 1.27(3)] with a bond angle of 121.10°(4.0) (table S4). Asp_CAT_ is present in two different conformations, a proximal position, as seen in the as-isolated structures of *Br*^2D^NiR, and a gatekeeper position that was first reported in an atomic resolution study for *Ac*NiR (*16*). This is the first observation of a gatekeeper position in a blue NiR, allowed by the 1 Å resolution of the structure with increased confidence provided by unrestrained SHELX refinement. The proximal and gatekeeper positions correlate well with the two conformations of bound nitrite (bent top-hat and top-hat), with refined Asp_CAT_ occupancies of (0.60) and (0.40) for proximal and gatekeeper positions, respectively. In addition, two conformations of the main-chain oxygen atom on residue Leu^100^ (named here for clarity as O_P_ and O_G_), located in the Asn^90^ channel allow a suitable hydrogen bond to be formed with a partial-occupancy water (W_G_) that is now observed instead of the second carboxyl oxygen (O^δ1^) atom of Asp_CAT_ proximal, with each coinciding with the presence and occupancy of the gatekeeper conformation (Fig. 3, A and C).

The position of W_G_ allows a suitable hydrogen bond to form with top-hat nitrite O^2^ atom (2.73 Å), but O^δ2^ atom of proximal Asp_CAT_ is situated too close (1.92 Å), emphasizing that proximal Asp_CAT_ and top-hat nitrite ligand are unlikely to be present at the same time. The proximal Asp_CAT_ O^δ2^ atom can form weak hydrogen bonding with the bent top-hat nitrite O^2^ atom (3.42 Å). Analysis of the carboxyl groups bond lengths (O^δ1^─C^γ^ and O^δ2^─C^γ^) in these conformations suggests that they are both negatively charged [the values are 1.25(3) and 1.26(3) for gatekeeper and 1.25(2) and 1.26(2) for the proximal position] (table S4). A partial-occupancy water (W5) present in the vicinity of T2Cu in line with O^1^ atoms in both conformations of nitrite is likely to be an important factor in stabilizing the substrate. Another notable feature of this structure is the visible rotation of the His_CAT_ imidazole ring toward the hydroxyl oxygen atom (O^ϒ1^) of Thr^275^ with equal hydrogen bonds for His_CAT_ N^δ1^ atom (~2.7 Å) with both Thr^275^ and Glu^274^ (Fig. 2B). The C─N─C angles associated with N^δ1^ and N^ε2^ atoms of His_CAT_ are 109.1°(5) and 109.2°(9), respectively, which corresponds to full residue protonation (fig. S1C and table S4) (*31*). The N^ε2^ atom of His_CAT_ is positioned within hydrogen bonding distance (3.06 Å) to bent top-hat nitrite O^2^ atom only (Fig. 3D), and W6, bridging His_CAT_ with Asp_CAT_, is seen in two positions correlating with proximal and gatekeeper positions of Asp_CAT_. The waters around the gatekeeper conformation of Asp_CAT_ are disordered, with multiple conformations associated with the two conformations of Asp_CAT_.

### XFEL FRIC structure of nitrite-bound enzyme at 1.3 Å resolution

As many or all diffraction images are collected from the same crystal volume in an SRX structure determination, nitrite-soaked CuNiR crystals undergo radiation-induced chemistry and turnover using electrons generated from the x-rays. To obtain a structure of nitrite-bound *Br*^2D^NiR FRIC structures, we obtained a 1.3 Å XFEL structure of *Br*^2D^NiR in its nitrite-bound state [confirmed by UV/vis microspectrophotometry on one of the crystals before irradiation at SACLA (fig. S2)] by using the SF-ROX technique (*33*) at 100 K. In SF-ROX, a single diffraction image is obtained from a single shot of <10-fs XFEL pulse per spot of a crystal, ensuring that data are captured before any x-ray–induced photochemistry can occur.

The most notable observation in this substrate-bound XFEL FRIC structure was the nitrite molecule at the T2Cu^2+^ site in a single side-on conformation with all three atoms O^1^, N, and O^2^ binding by almost identical distances of 2.12, 2.02, and 1.92 Å at an occupancy of 0.7 (Fig. 2B). This is the first time that a single side-on nitrite has been observed at the T2Cu^2+^ site in an XFEL FRIC structure from any CuNiR. The positions of O^1^ and O^2^ atoms are very close to top-hat conformation of nitrite in the atomic-resolution SRX structure. The side-on nitrite in this FRIC structure is now in an ideal position to the full-occupancy proximal Asp_CAT_, with its O^2^ atom 2.25 Å away from Asp_CAT_ O^δ2^ atom (Fig. 3, B and E). Half-occupancy water W4 is now bonded with O^1^ atom of nitrite. His_CAT_ has its imidazole ring and its N^δ1^ atom rotated toward residue Glu^274^ carbonyl oxygen again with a shorter hydrogen bond of 2.52 Å in comparison with the atomic-resolution nitrite-bound SRX structure (Fig. 2B) and both structures of *Br*^2D^NiR in its as-isolated state. N^ε2^ atom of His_CAT_ is positioned 3.17 Å away from side-on nitrite O^2^ atom and 2.94 Å from W6.

### Low-dose (0.5 MGy) nitrite-bound SRX structure at 1.48 Å resolution

To confirm that the structural differences of His_CAT_ in our nitrite-bound structures were due to x-ray induced electron transfer after substrate binding in the 1 Å SRX structure, we obtained a low-dose [0.5 MGy] SRX dataset to 1.48 Å resolution. It showed a single “L-shaped” nitrite with coordination distances to T2Cu of 2.33, 1.78, and 1.92 Å for O^1^, N, and O^2^ atoms, respectively (fig. S5A). Asp_CAT_ is again present in both its main (occupancy, 0.6) and distorted conformation (occupancy, 0.2) of its proximal position, as seen in the as-isolated SRX structure. Distorted conformation O^δ2^ atom is 1.95 Å away from the O^2^ atom of nitrite, and main conformation O^δ2^ atom is 2.36 Å away. W6 is seen in two positions correlating with main proximal and distorted proximal positions of Asp_CAT_. No change in His_CAT_ position is observed in comparison with the XFEL FRIC structure (fig. S5B), providing clear evidence that rotation of the His_CAT_ imidazole ring toward the hydroxyl oxygen (Oϒ^1^) atom of Thr^275^ seen in the 1 Å SRX structure is due to the initiation of the catalytic reaction induced by x-ray–generated electrons reaching the nitrite-bound T2Cu.

### Enzymatically generated nitrosyl species and its SRX structure at 1.19 Å resolution

We have used a new approach to obtain the catalytically formed NO-bound species by chemically inducing NiR turnover in the crystals. The nitrite soaking of crystals that ensured full nitrite occupancy as demonstrated above was followed by a time-dependent soak with the strong reductant dithionite to initiate the catalytic reaction by chemical reduction of T1Cu with the expectation of capturing a NO-bound state through the catalytic conversion of bound nitrite to nitric oxide, with NO remaining bound to T2Cu as an essential reaction intermediate. The total reduction of T1Cu was monitored by obtaining UV/vis microspectrophotometry data on the colorless crystals (fig. S2).

**Fig. 4:**
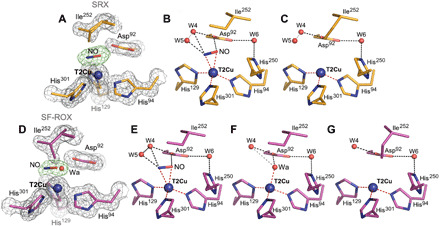
T2Cu site in NO-bound/T1Cu-reduced *Br*^2D^NiR: SRX v SF-ROX structures. (**A**) SRX NO-bound structure (1.19 Å; orange) has two conformations of the active site: (**B**) one with NO present and (**C**) with three coordinated T2Cu site. (**D**) In the 1.3 Å XFEL FRIC structure (magenta), there are three alternative conformations of the T2Cu site: (**E**) one with NO, (**F**) second with water, and (**G**) third with three coordinated T2Cu site where NO has vacated the T2Cu site with alternative Ile^252^ conformation of CD atom flipping down to fill the space. Water molecules are shown as red spheres, and T2Cu is shown as a blue sphere. Distances for possible hydrogen bonding are shown as black dashed lines; gray dashed lines represent unlikely bonding due to steric restraints, and coordination distances to T2Cu are shown as red dashed lines. OMIT *F*_o_ − *F*_c_ electron density maps around T2Cu ligands are contoured at 5σ level and colored green. Gray 2*F*_o_ − *F*_c_ electron density map is contoured at 1σ level.

A 1.19 Å structure from a reductant-treated nitrite-soaked crystal allowed us to perform unrestrained refinement with SHELX. A NO molecule could be placed at the T2Cu site with occupancy (0.73) refined by SHELX (Fig. 4, A and B). It is clearly coordinated in a side-on manner, with slightly asymmetric distances of 2.17(4) and 2.08(3) Å for Cu─N and Cu─O, respectively. The O atom of NO is positioned 2.48 Å away from O^δ2^ of Asp_CAT_ in proximal position. Analysis of the carboxyl group bond lengths of Asp_CAT_ 1.24(3) Å for O^δ1^─C^γ^ and 1.28(3) Å for O^δ2^─C^γ^ suggests that Asp_CAT_ could be protonated at O^δ2^ (table S4). W6 bridging water is connecting O^δ1^ atom of Asp_CAT_ with His_CAT_ N^ε2^ at ~2.9 Å. His_CAT_ imidazole is in its usual position, forming a hydrogen bond between His_CAT_ N^δ1^ and Glu^274^ carbonyl oxygen at 2.62 Å, similar to the as-isolated SRX structure. His_CAT_ is only protonated at N^δ1^ atom, as C─N─C angle associated with N^δ1^ atom is 113.5°(2.4), while similar angle associated with N^ε2^ is much smaller 105.0°(2.2), which corresponds to nonprotonated state (fig. S1A and table S4) (*31*).

### Enzymatically generated NO-bound XFEL FRIC structure determined at 1.3 Å resolution

We were successful in obtaining a damage-free XFEL structure of *Br*^2D^NiR in its NO-bound state at 100 K using the SF-ROX technique (*33*) using the dithionite-reduced nitrite-soaked crystals. We were able to capture three distinct species with structures representing stages of bound NO (resulting from in situ nitrite reduction) and its dissociation under these turnover conditions. The data are consistent with a mixture of enzymatically produced NO-bound, a ligand-free, and water-ligated T2Cu states (Fig. 4D). In the first conformation of the active site, NO was modeled bound to the T2Cu site with an occupancy of 0.4 (Fig 4, D and E), and it was coordinated to T2Cu with even greater asymmetry in the distances of 2.47 and 2.12 Å for Cu─N and Cu─O, respectively, with the O atom forming a hydrogen bond with O^δ2^ atom of proximal Asp_CAT_ (2.56 Å) (Fig. 4E). In the second species, a single water molecule with occupancy of 0.3 was assigned at the T2Cu site (Fig. 4, D and F). This water coordinated to T2Cu at a distance of 2.07 Å is also able to hydrogen bond to O^δ2^ of Asp_CAT_ by 2.16 Å (Fig. 4F). In the third species, the active site had a previously unseen conformation of the conserved Ile_CAT_ residue (Ile^252^) where the side chain is flipped to occupy the space in the T2Cu active-site cavity with an occupancy of 0.25 (Fig. 4, D and G). Again, because of steric restraints, this conformation can only exist when neither the NO nor the water ligand is present at the active site (Fig. 4G) and represents a new intermediate representing the status of catalytic Cu where NO is released but the T2Cu is tricoordinate and has not yet acquired the water ligand required for returning to the resting state. This Ile position is similar to Cu^+^(His)_3_ coordination formed on chemical reduction of the resting state of CuNiR, where the T2Cu is devoid of the water ligand, resulting in loss of activity (*29*, *35*). As in the SRX NO-bound structure, the His_CAT_ imidazole ring is rotated toward Glu^274^ carbonyl oxygen atom at 2.61 Å (Fig. 2C). The reduction of T1Cu site, as confirmed by the absence of bands in optical spectra (fig. S2), results in slight elongation of electron density of the T1Cu atoms and surrounding residues, consistent with subtle changes expected to accompany Cu^2+^/Cu^+^ redox change for cupredoxin.

## DISCUSSION

### Accuracy needed for defining chemistry of catalytic pocket residues in resting and nitrite-bound enzyme

It has been shown previously that at atomic resolution, the bond lengths’ e.s.d.s. can be defined with an accuracy better than 0.008 Å (*36*). Such accuracy allowed us to determine the protonation states of some of the catalytically important residues from an analysis of the bond lengths in the catalytic coordination sphere. To remove influence of the crystallographic dictionary restraints on the structure, we used unrestrained refinement at the final step in SHELXL and determined e.s.d.s. for each of the bond lengths, including T1Cu and T2Cu coordinating spheres (tables S2 and S3). We also evaluated the protonation states of the catalytically important Asp_CAT_ (Asp^92^) and His_CAT_ (His^250^) residues. When aspartate is nonprotonated, the bond lengths between C^γ^─O^δ1^ and C^γ^─O^δ2^ are expected to be equal to 1.256(15) Å; upon protonation, one bond will increase to 1.310(17) Å and the other will become a double bond with a distance of 1.210(16) Å [data are from the Cambridge Structural Database (www.ccdc.cam.ac.uk)] (fig. S1, D and E). Analysis of the bond lengths for the carboxyl group of Asp_CAT_ in the SRX as-isolated structure at 1.1 Å resolution is consistent with the protonation, only when this residue is in the distorted position. In contrast, in the SRX nitrite-bound structure at 1 Å atomic resolution, Asp_CAT_ is negatively charged in both gatekeeper and proximal conformations. The SRX NO-bound structure at 1.19 Å resolution shows deviation in the bond lengths [1.24(3) Å for O^δ1^─C^γ^ and 1.28(3) Å for O^δ2^─C^γ^)] and possible protonation of O^δ2^ atom. The double conformations of Asp_CAT_ in both as-isolated and nitrite-bound SRX structures result in slightly reduced accuracy but are somewhat compensated by low-temperature factors and ~70% occupancy of the main conformation of Asp_CAT_ associated with two waters present in the active site (Fig. 1, A and B, and table S4). The analysis of bond angles for the His_CAT_ residue imidazole ring in SRX structures (table S4) suggests its full protonation only in the 1 Å resolution SRX nitrite-bound structure, where rotation of the imidazole ring is seen.

### Role of distorted proximal conformation of Asp_CAT_

The lower distorted proximal rotamer of Asp_CAT_ was first reported in the SF-ROX XFEL structure of the as-isolated green *Ac*NiR (*29*), where it was suggested to shorten the hydrogen bond between the O^δ2^ atom of Asp_CAT_ and the T2Cu coordinated W1, compared to main proximal position. However, as the same conformation in our structure appears to have an association only with the latent W3 within the T2Cu active site of SRX structure and also appears to be protonated, where its main conformation is not, suggesting a more important role. We may have either observed a flexible position of proximal Asp_CAT_, which has a role in chaperoning T2Cu waters from the substrate channel/Asn^90^ proton channel to their coordinated positions within the T2Cu site or have captured a unique conformation where a proton is soon to be abstracted by Asp_CAT_ to a T2Cu ligand in as-isolated state. As distorted conformation is also observed in the low-dose nitrite-bound structure (0.5 MGy), its role as a conformation prepped for proton abstraction is more probable.

### Mode of nitrite binding (side-on versus top-hat configuration) in NiR

The 1 Å SRX and 1.3 Å XFEL structures of nitrite-bound *Br*^2D^NiR allow a detailed comparison of nitrite binding in a blue CuNiR with those of green CuNiRs. It also helps to consolidate these high-resolution structural data to reach a consensus scheme. The two different conformations of the bidentate top-hat binding modes for nitrite in the SRX structure correlating with gatekeeper and proximal positions of Asp_CAT_ indicate that the mobility of this essential residue is critical in controlling the passage of the substrate and its anchoring in all CuNiRs (*16*, *29*, *37*, *38*). The top-hat vertical bidentate position can only be present with Asp_CAT_ in its gatekeeper position in *Br*^2D^NiR because of steric constraints. The single horizontal side-on position of nitrite with coordination of all atoms to a fully oxidized Cu^2+^ site of *Br*^2D^NiR has been seen for the first time in an XFEL FRIC structure of a CuNiR. The clear observation of full-occupancy side-on horizontal coordination of nitrite provides unambiguous evidence that the bidentate oxygen binding mode of nitrite to a fully oxidized T2Cu^2+^ in contrast to the monodentate binding being proposed previously (*39*). It also indicates that the conformational change from top-hat to side-on position is not a result of reduction (*29*). As indicated earlier, *Br*^2D^NiR has a more open substrate channel compared to other CuNiRs, resulting in more waters being present in the vicinity of the T2Cu in the nitrite-bound structure compared to the XFEL structures of green *Af*NiR and *Ac*NiR.

### Enzymatically produced copper nitrosyl species defined

Our new approach to obtain NO-bound intermediate generated in turnover of a nitrite-soaked crystals of a CuNiR provides insight into binding of NO that is enzymatically generated. The observation of an in situ produced copper nitrosyl species in both the SRX and XFEL FRIC structures at ~1.2 Å unambiguously demonstrates this as a catalytically relevant reaction intermediate, refuting the hypothesis that “the proton-coupled electron transfer (PCET) to the nitrite-bound T2Cu leads to reduction of nitrite to form NO, which is released without forming a Cu-nitrosyl species” (*22*–*24*). The side-on binding copper-nitrosyl coordination observed in all of the crystallographic structural studies irrespective of the organisms (*16*, *37*, *38*, *40*), including the current in situ enzymatically generated species, is at variance with “bent” or “end-on” binding modes suggested on the basis of synthesis of chemical compounds and computational studies (*15*, *25*, *27*, *41*–*44*) and needs to be taken into account if the synthetic biomimetic systems are to prove effective NiRs.

### Lower activity of *Br*^2D^NiR compared to *Ac*NiR and *Ax*NiR

Our as-isolated SRX and XFEL FRIC structure provides insight into the much lower catalytic efficiency of *Br*^2D^NiR compared to two of the well-studied two-domain CuNiRs represented by the blue (*Ax*NiR) and green (*Ac*NiR) subclasses (*21*). A notable feature of the as-isolated *Br*^2D^NiR compared with other CuNiRs is the presence of two water ligands simultaneously bound to T2Cu confirmed by both atomic-resolution SRX and XFEL FRIC structures. The XFEL FRIC structure clearly shows two full-occupancy waters coordinated to T2Cu. This contrasts from other CuNiRs where a single water is observed and is the likely origin of lower specific activity of this enzyme. We note that two ligands were also seen coordinated to T2Cu (partial waters or chloride and water) in CuNiR from *Geobacillus thermodenitrificans* (*Gt*NiR), but contaminants close to the active site, including a sodium ion and/or the coordinated chloride ion and an extra copper ion at His_CAT_, made the functional significance of these observations less clear (*39*, *45*). A high-resolution neutron crystallographic structure of *Gt*NiR suggested that one of these waters is actually a hydroxide ion (OH^−^) in its resting state (*23*), but again, the study revealed excess Cu (at least four) in the structure. Another case, where an extra water is seen close to the T2Cu site of a two-domain blue CuNiR, is on an F306C mutation of *Ax*NiR, a surface residue at the mouth of the substrate access channel (*46*) some 12 Å from the T2Cu site. This mutation also showed considerably decreased catalytic efficiency, assigned to the second water, making nitrite binding energetically less favorable because of extra hydrogen bonding to the water bound to the T2Cu. Similarly, to this mutant, *Br*^2D^NiR has an alanine residue at the mouth of the substrate channel instead of a bulky phenylalanine seen in other well-studied two-domain CuNiRs (*Ax*NiR, *Ac*NiR, and *Af*NiR). This results in the active site being more accessible to solvent and is likely to be the reason for an extra water bound at T2Cu active site of as-isolated structure. The increased accessibility to solvent and two strongly coordinated waters may contribute to a much lower NiR catalytic activity in *Br*^2D^NiR when compared to *Ax*NiR and *Ac*NiR. Activity data for *Gt*NiR have not been published.

### Proposed catalytic reaction

**Fig. 5:**
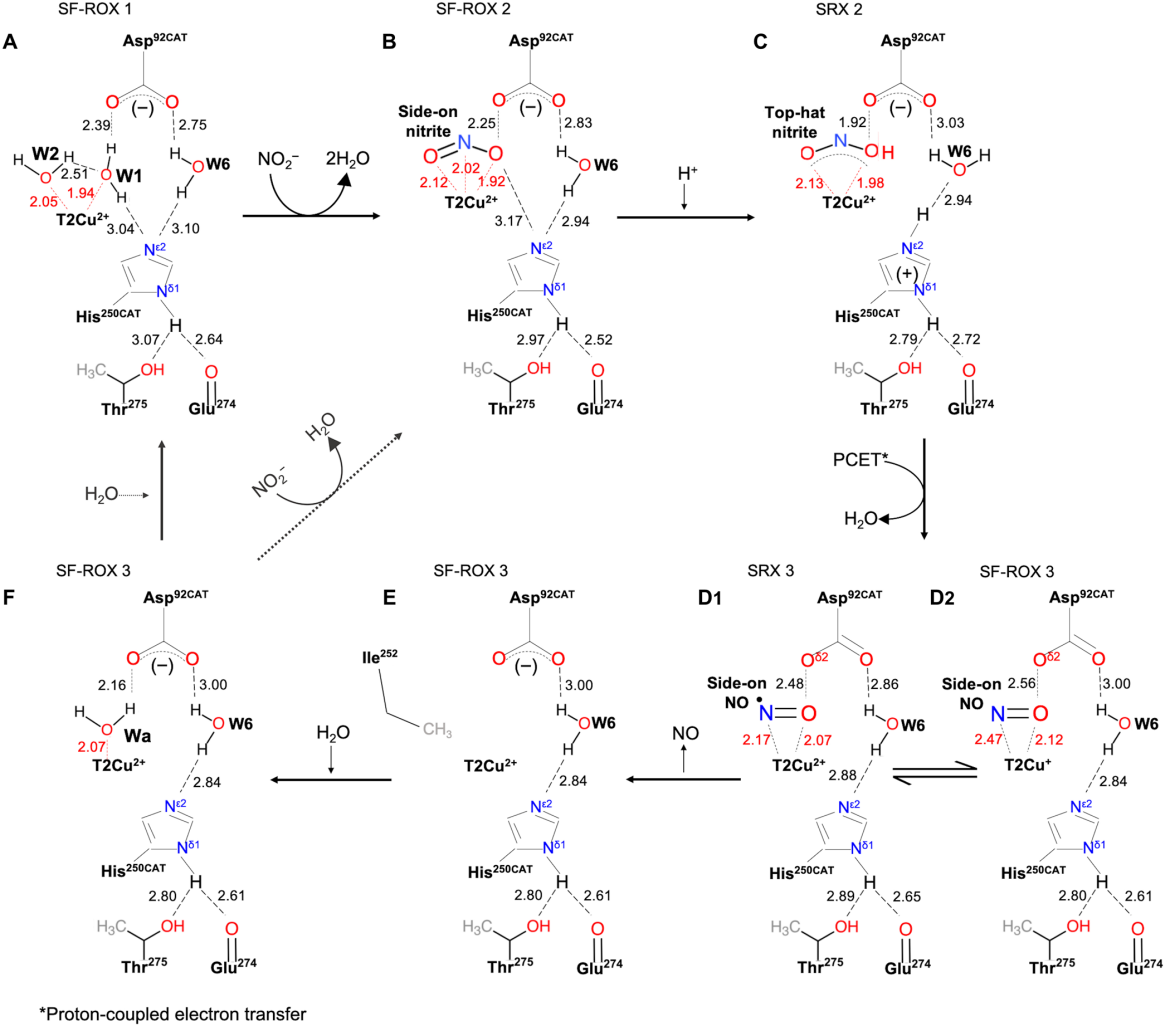
Proposed structure-based catalytic mechanism of *Br*^2D^NiR. Solid arrows show the reaction sequence based on the structures of as-isolated and ligand-bound species of *Br*^2D^NiR described here (**A** to **F**). The further reaction of species (F) in CuNiRs that have a single water ligand of T2Cu in the resting state is shown as a dashed line. (A) Two waters bound in as-isolated SF-ROX structure 1 and in conformation 1 of as-isolated SRX structure 1. (B) Side-on nitrite bound in nitrite-bound SF-ROX structure 2. (C) Intermediate state with top-hat nitrite bound in nitrite-bound SRX structure 2. (D1) and (D2) represent two slightly different NO bound conformations in the SF-ROX 3 (D2) and SRX 3 (D1) structures; in D1, N and O of NO are almost equidistant. (E) Tricoordinate T2Cu site in NO-bound SF-ROX structure 3 where NO is no longer at the T2Cu site, resulting in the flipping of Ile^252^ into the space vacated from loss of fourth ligand. (F) Single water bound in NO-bound SF-ROX structure 3 where the water has been captured by the T2Cu site following the release of NO. The step from (F) to (B) represents the well-studied CuNiRs, such as *Ac*NiR, *Af*NiR, and *Ax*NiR, while (F) to (A) represents the return of *Br*^2D^NiR to its resting state.

In addition to being a substrate in the reaction catalyzed by CuNiRs, protons also have a role in gating electron transfer from the T1Cu site to the catalytic T2Cu site, both in the presence and absence of nitrite (*20*, *47*). Our SHELX analysis of substrate- and product-bound intermediates allows the protonation states of two essential active site residues, Asp_CAT_ and His_CAT_, to be defined throughout the catalytic cycle, allowing us to refine the proposed mechanism for CuNiRs. We suggest the following catalytic mechanism based on previous structural and biophysical studies of CuNiR and incorporating the new data presented here: The resting state of the oxidized enzyme has water (two waters in the case of *Br*^2D^NiR) bound to T2Cu site (Fig. 5A). Asp_CAT_ is in the proximal position, and neither Asp_CAT_ nor His_CAT_ N^ε1^ is protonated as seen in both SRX and XFEL FRIC structures and neutron crystallography of *Ac*NiR, the only neutron study where only T1/T2Cu were present and no exogenous ligand was bound to the T2Cu (*29*). Initially, nitrite binds side-on with all three atoms ligated to the T2Cu site by displacement of the water ligand(s), as seen in the nitrite-bound XFEL FRIC structure (Fig. 5B), followed by protonation of His_CAT_ and the proposed Cu-NOOH intermediate (Fig. 5C), triggering proton-coupled electron transfer from T1Cu site and reduction of the T2Cu site. This then leads to reduction of nitrite via OH─N─O bond breakage to nitric oxide and release of water (Fig. 5, D1/D2). The release of water leaves NO coordinated in side-on manner (Fig. 5, D1/D2), consistent with a rigorous Density functional study (*48*). Release of nitric oxide leaves the T2Cu site tricoordinate (Fig. 5E), ready to capture a water molecule and return to the resting state (Fig. 5F) and enter the next cycle of nitrite binding and reduction.

## CONCLUSIONS

We have undertaken a comprehensive structural study of a new two-domain blue CuNiR from *Bradyrhizobium* sp. ORS 375 using SR and XFEL to obtain the most accurate (SR structures at resolutions of ~1 Å, enabling unrestrained SHELX refinement, not undertaken for any CuNiR). We determined damage-free (XFEL) FRIC structures, in the as-isolated, substrate-bound, and product-bound states, to high resolutions of 1.3 Å. For all of the structures, optical spectra were collected on single crystals. This combination of structural approaches has allowed characterization of the stable nitrosyl intermediate with side-on binding, unequivocally refuting the hypothesis that the proton-coupled electron transfer to the nitrite-bound T2Cu leads to reduction of nitrite to form NO, which is released without forming a Cu-nitrosyl species. We demonstrate that during enzyme turnover, the mobility of Asp_CAT_ to adopt the gatekeeper and proximal positions is critical in controlling the passage of the substrate and its anchoring at the T2Cu site in both blue and green CuNiRs. The single horizontal side-on position of nitrite with coordination of all atoms to T2Cu^2+^ site of a fully oxidized of *Br*^2D^NiR enzyme crystals has been seen for the first time in any XFEL FRIC structure of a CuNiR. The ability to enzymatically generate the product in situ allowed us to capture “the act of product release” that leaves the T2Cu site tricoordinate. Our results illustrate that the combined approach of very-high-resolution SRX structures with unrestrained SHELX refinement together with FRIC structures on crystals using XFEL in well-defined states supported by single crystal spectroscopy has wider applicability to structural enzymology. It is likely to be particularly helpful in elucidating catalytic mechanisms of metalloenzymes, which would gain further boost from the availability of higher-energy x-ray pulses from XFEL that should allow even higher resolution structures approaching ~1 Å.

## MATERIALS AND METHODS

### *Br*^2D^NiR expression and purification for both as-isolated structures and atomic-resolution SRX nitrite-bound structure

Recombinant plasmid for wild-type (WT) two-domain *Br*NiR [pET-26b(+)-^WT^*Br*^2D^NiR-TEV-6xHis] was purchased from GenScript. *Escherichia coli* BL21 (DE3) cells harboring the plasmid were added to 500 ml of LB broth supplemented with kanamycin (50 μg/ml) and incubated at 37°C until an optical density at 600 nm of ~0.6 was reached. Overexpression of *Br*^2D^NiR was induced with 500 μM isopropyl-β-d-thiogalactopyranoside with 1 mM CuSO_4_ added, followed by incubation of cell culture for 18 hours at 180 rpm before cells were harvested by centrifugation. Cell pellet was collected and resuspended in 100 mM tris-HCl buffer (pH 8.0), containing 500 mM NaCl (buffer A) supplemented with lysozyme (50 μg/ml) and protease inhibitor and disrupted by sonication. Cleared cell lysate, collected by centrifugation, was dialyzed overnight against 1 mM CuSO_4_ in buffer A at 4°C. Blue lysate was loaded onto a HisTrap HP Ni affinity column (GE Healthcare), equilibrated with 10 mM imidazole in buffer A. Protein was eluted with 250 mM imidazole in the same buffer and consequently dialyzed overnight against 100 mM tris-HCl, 500 mM NaCl, and 10% (v/v) glycerol (pH 8) (buffer B) before loading onto a HiLoad Superdex 200 16/600 pg size exclusion chromatography (SEC) column (GE Healthcare) equilibrated with the same buffer B. A final overnight dialysis against 1 mM CuSO_4_ in buffer B at 4°C was required to achieve full copper loading of the T2Cu site. Before crystallization, protein was buffer-exchanged into 10 mM Hepes (pH 6.5).

### *Br*^2D^NiR expression and purification for all other structures

As above but with the following changes, cleared cell lysate, collected by centrifugation, was loaded onto the same HisTrap HP column, equilibrated with 10 mM imidazole in buffer A. Protein was eluted with 250 mM imidazole in the same buffer, dialyzed against buffer B overnight, and then loaded onto to the same SEC column equilibrated with the same buffer. Elution was again dialyzed overnight against 1 mM CuSO_4_ in buffer B at 4°C and applied to a SEC column, as before. A final overnight dialysis was required to achieve full copper loading of the T2Cu site, with overnight dialysis against 1 mM CuSO_4_ in buffer B at 4°C. The solution was applied to a SEC column and then buffer-exchanged into 10 mM Hepes (pH 6.5) before crystallization.

### Crystallization and crystal treatment

All *Br*^2D^NiR crystals were grown using the vapor diffusion hanging drop method at room temperature, with a protein concentration of 30 mg/ml mixed 1:1 with reservoir solution composed of either 1.6 or 1.8 M ammonium sulfate with 50 mM Hepes buffer (pH 5 or pH 5.5, respectively). Crystals in space group *P*2_1_3 grew within a few days.

#### Synchrotron-radiation crystallography

For SRX as-isolated experiment, the crystal was soaked into a cryoprotectant solution composed of 3.6 M ammonium sulfate and 50 mM Hepes buffer (pH 5.5), while SRX nitrite-bound crystals were soaked in a solution composed of 100 mM NaNO_2_, 2.4 M ammonium sulfate, and 50 mM Hepes buffer (pH 5.5) for 30 s and then transferred into a cryoprotectant solution composed of 100 mM NaNO_2_, 3.6 M ammonium sulfate, and 50 mM Hepes buffer (pH 5.5) before both were cryocooled by plunging into liquid nitrogen and stored in pucks for data collection. Crystals for SRX NO-bound structure was soaked in a nitrite solution composed of 200 mM NaNO_2_, 2.5 M ammonium sulfate, and 50 mM Hepes buffer (pH 5.5) for 30 s and then transferred into a cryoprotectant solution composed of 200 mM NaNO_2_, 3.3 M ammonium sulfate, 20.3% sucrose, and 50 mM Hepes buffer (pH 5.5) before lastly being soaked into a dithionite containing solution composed of 100 mM sodium dithionite, 3.3 M ammonium sulfate, 20.3% sucrose, and 50 mM Hepes buffer (pH 5.5) until the crystals became completely colorless before being cryocooled by plunging into liquid nitrogen and stored in pucks for data collection.

#### Serial femtosecond rotational crystallography

For SF-ROX as-isolated structure, crystals were soaked into a cryoprotectant solution composed of 3.3 M ammonium sulfate, 20.3% sucrose, and 50 mM Hepes buffer (pH 5.5). SF-ROX nitrite-bound crystals were soaked into a nitrite-containing solution composed of 200 mM NaNO_2_, 2.5 M ammonium sulfate, and 50 mM Hepes buffer (pH 5.5) for 30 s and then transferred for 30 s into a cryoprotectant solution composed of 200 mM NaNO_2_, 3.3 M ammonium sulfate, 20.3% sucrose, and 50 mM Hepes buffer (pH 5.5) for the same time before being cryocooled by plunging into liquid nitrogen. SF-ROX NO-bound crystals were prepared in the same way as for the SRX NO-bound structure.

### Data collection, processing, and refinement

#### Synchrotron-radiation crystallography

Crystallographic data for atomic-resolution as-isolated and nitrite-bound structures were collected at 100 K using a DECTRIS PILATUS3 6M detector on I03 beamline at Diamond Light Source, UK at a wavelength of 0.97634 and 0.86999 Å, respectively. For NO-bound, data collection was carried out on BL41XU at SPring-8 using a DECTRIS EIGER X 16M detector at a wavelength of 0.80000 Å. All Diamond datasets were processed using DIALS (*49*) with xia2 (*50*) through Diamond’s automated processing pipeline. Data at SPring-8 were processed using XDS (X-ray Detector Software) (*51*) and CCP4 (The Collaborative Computational Project Number 4) packages using the automated data processing pipeline KAMO (*52*). Using structure 6THF as a starting model, SRX as-isolated, nitrite-bound, and NO-bound structures were refined with REFMAC5 (*53*) in the CCP4 program suite (*54*) with anisotropic B-factors and riding hydrogen atoms. For atomic-resolution structures, as-isolated, nitrite-bound, and NO-bound SRX, REFMAC5 refinement was followed up by refinement with SHELX-97 (*28*), where refinement of anisotropic B-factors, hydrogen positions, occupancies of the double conformation of the side chains, and T2Cu ligands was used. At the final stage of the refinement, one cycle of unrestrained block-matrix least-squares refinement was implemented to estimate the SDs (e.s.d.s.) of coordinates and derived parameters (bond lengths and angles). Each block consisted of 54 residues with 5 residue overlap. The information on the Cu ligands bonding with e.s.d.s. is shown in tables S2 and S3. Manual model rebuilding was performed using Coot (*55*), with ligands added to weighted election density maps and waters added after each stage of refinement. Data collection and refinement statistics are shown in table S1.

#### Serial femtosecond rotational crystallography

For each dataset, a total of 40 (as-isolated), 59 (nitrite-bound), and 55 (NO-bound) crystals of sizes ranging from 0.4 to 0.7 mm were frozen and used for collection. Data collection was performed on BL2/EH3 at SACLA at 100 K, as described previously (*33*), with the following parameters of XFEL pules: pulse duration (<10 fs), pulse energy (10 keV), and beam size at sample position [6.2 μm (*H*) by 2.5 μm (*V*)] for all. The crystals were rotated 0.1° and translated 50 μm between each shot. The diffraction images were collected on a MX300-HS detector (Rayonix) with a camera length of 90 mm and a pixel size of 78.2 μm. The same data collection and processing procedure were applied for all three datasets; twenty-eight crystals were selected for processing for nitrite-bound, and all crystals were selected for processing for both as-isolated (40) and NO-bound (55). Indexing and integration were performed using CrystFEL version 0.6.3 (*56*) with the inner, middle, and outer integration radii set to of 6, 7, and 8 pixels respectively. The spot-finding algorithm peakfinder8 was used, and indexing was carried out using DirAx (*57*) and Mosflm (*58*). After the resolution of the indexing ambiguity using ambigator, Bragg intensities were merged using the Monte Carlo integration with linear frame scaling. When merging, the high-resolution limit (*d*^−1^) in each pattern was extended by 1.7 nm^−1^ (as-isolated and nitrite-bound) or 1.9 nm^−1^ (NO-bound) using --push-res option.

In both cases, hydrogen bond contact distances were measured using CONTACT in the CCP4 program suite (*54*) and manually in Coot. All distances are listed in table S5.

### UV/vis microspectrophotometry of samples in crystalline state

Optical absorption spectra for single uncollected crystals prepared for each SF-ROX dataset were obtained at 100 K using a fiber-optic microspectrophotometer with linear charge-coupled device array detector (Ocean Optics, SD2000).

## Supplementary Material

http://advances.sciencemag.org/cgi/content/full/7/1/eabd8523/DC1

Adobe PDF - abd8523_SM.pdf

An unprecedented insight into the catalytic mechanism of copper nitrite reductase from atomic-resolution and damage-free structures

## SUPPLEMENTARY MATERIALS

Supplementary material for this article is available at http://advances.sciencemag.org/cgi/content/full/7/1/eabd8523/DC1

## Appendix

View/request a protocol for this paper from *Bio-protocol*.
