# Supplementary material for: An unprecedented insight into the catalytic mechanism of copper nitrite reductase from atomic-resolution and damage-free structures
Source: Sci Adv. 2021 Jan 1;7(1):eabd8523. doi: 10.1126/sciadv.abd8523 (PMC7775769; doi:10.1126/sciadv.abd8523)
Supplement: http://advances.sciencemag.org/cgi/content/full/7/1/eabd8523/DC1 [file supp_7_1_eabd8523__index.html]

Science Advances | Science AdvancesAAASSearchScience AdvancesMenu

## Supplementary Materials

# An unprecedented insight into the catalytic mechanism of copper nitrite reductase from atomic-resolution and damage-free structures

Samuel L. Rose, Svetlana V. Antonyuk, Daisuke Sasaki, Keitaro Yamashita, Kunio Hirata, Go Ueno, Hideo Ago, Robert R. Eady, Takehiko Tosha, Masaki Yamamoto, S. Samar Hasnain

Download Supplement

**This PDF file includes:**

- Supplementary Materials and Methods
- Figs. S1 to S5
- Tables S1 to S5
- References

**Files in this Data Supplement:**

- Adobe PDF - abd8523\_SM.pdf
