# Supplementary material for: An unprecedented insight into the catalytic mechanism of copper nitrite reductase from atomic-resolution and damage-free structures
Source: Sci Adv. 2021 Jan 1;7(1):eabd8523. doi: 10.1126/sciadv.abd8523 (PMC7775769; doi:10.1126/sciadv.abd8523)
Supplement: http://advances.sciencemag.org/cgi/content/full/7/1/eabd8523/DC1 [file supp_7_1_eabd8523__1.pdf]

Science Advances
AAAS

advances.sciencemag.org/cgi/content/full/7/1/eabd8523/DC1

Supplementary Materials for

An unprecedented insight into the catalytic mechanism of copper nitrite reductase from atomic-resolution and damage-free structures

Samuel L. Rose, Svetlana V. Antonyuk, Daisuke Sasaki, Keitaro Yamashita, Kunio Hirata, Go Ueno, Hideo Ago, Robert R. Eady, Takehiko Tosha, Masaki Yamamoto*, S. Samar Hasnain*

*Corresponding author. Email: yamamoto@riken.jp (M.Y.); s.s.hasnain@liverpool.ac.uk (S.S.H.)

Published 1 January 2021, *Sci. Adv.* **7**, eabd8523 (2021)
DOI: 10.1126/sciadv.abd8523

This PDF file includes:

Supplementary Materials and Methods
Figs. S1 to S5
Tables S1 to S5
References

Supplementary Materials and Methods

Low dose (0.5 MGy) nitrite-bound SRX structure at 1.48 Å resolution

Crystallization and crystal treatment

Crystal used for low dose (0.5 MGy) nitrite-bound SRX structure was grown in 1.6 M Ammonium sulfate and 50 mM HEPES buffer (pH 5.5) and soaked in same nitrite and cryoprotectant solution as the damage-free nitrite-bound structure: 200 mM NaNO2, 2.5 M Ammonium sulfate and 50 mM HEPES buffer (pH 5.5) for 30 seconds and then transferred for 30 seconds into a cryoprotectant solution consisted of 200 mM NaNO2, 3.3 M Ammonium sulfate, 20.3% sucrose and 50 mM HEPES buffer (pH 5.5)) for the same time before being cryocooled by plunging into liquid nitrogen.

Data collection, processing and refinement

Data collection was carried out on BL41XU at SPring-8 using a Dectris EIGER X 16M detector at a wavelength of 0.80000 Å, with a total dose of 0.5 MGy (calculated using RADDOSE-3D(*59*)) set as a parameter before collection took place. Data at SPring-8 was processed using XDS(*51*) and CCP4 packages(*54*) using the automated data processing pipeline: KAMO(*52*). Refinement was performed with REFMAC5(*53*). Manual model rebuilding was performed using Coot(*55*) with ligands added to weighted election density maps and waters added after each stage of refinement. Data collection and refinement statistics are shown in Table S1.

Figure S1

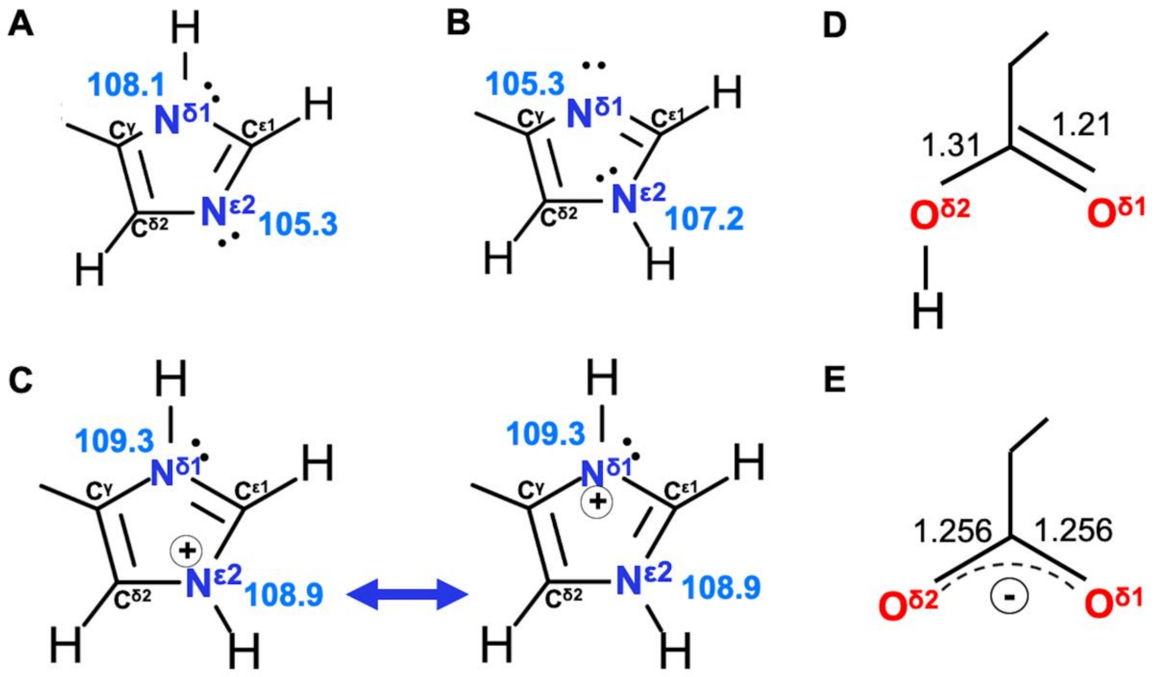

Fig. S1. Electronic states of the imidazole rings and Aspartates.

(**A**) Neutral charge - $N^{\delta 1}$ atom is protonated. (**B**) Neutral charge - $N^{\varepsilon 2}$ atom is protonated. (**C**) Positively charged imidazole ring with both N atoms protonated: the value of the C-N-C angle based on analysis of the CSD database is shown in blue. (**D**) Positively changed aspartate. (**E**) Negatively charged aspartate. Bond lengths values and data are from the Cambridge Structural Database (CSD; http://www.ccdc.cam.ac.uk)(*36*, *32*).

Figure S2

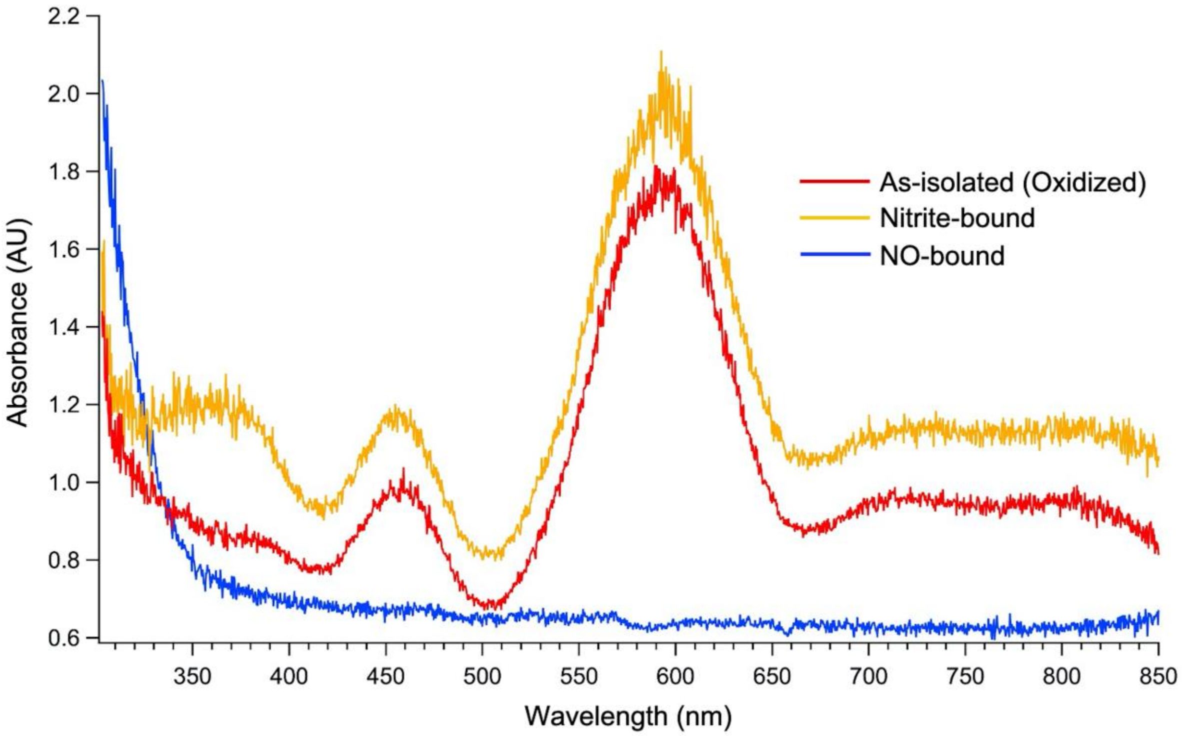

Fig. S2. UV/vis absorption spectra from *Br*^{2D}NiR crystals prior to X-ray exposure at SACLA.

The band at ~590 and 455nm clearly shows that T1Cu is in Cu^{2+} state. In nitrite soaked crystal (yellow orange), the band at ~360 is from nitrite charge transfer.

Figure S3

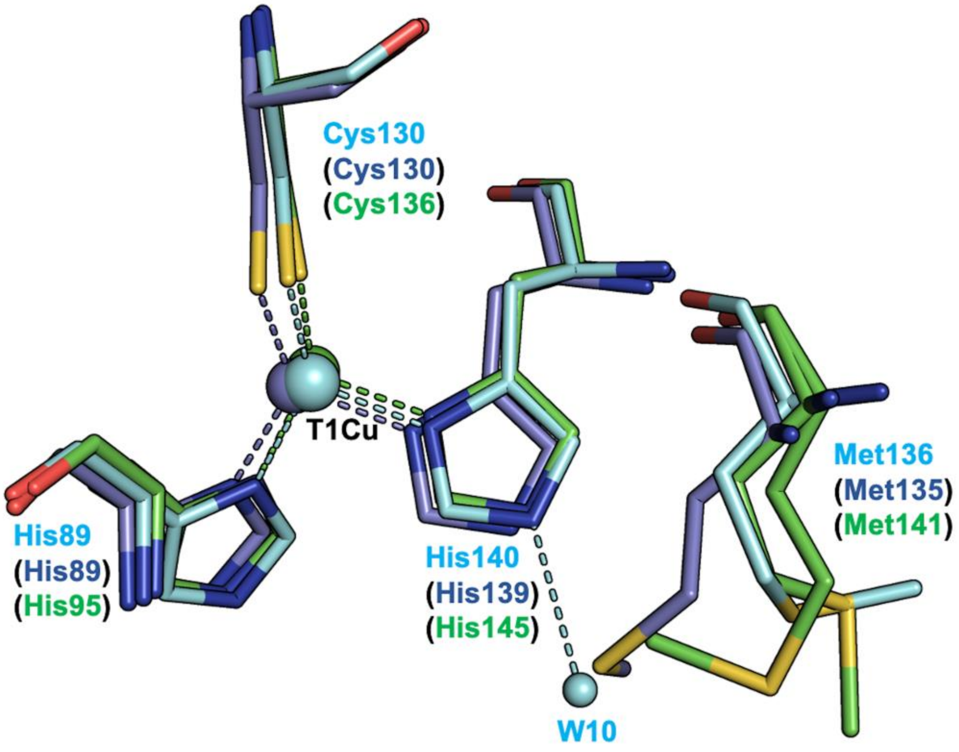

Fig. S3. Superposition of the T1Cu site in fully oxidized XFEL FRIC structures of *Br*^{2D}NiR, blue *Ax*NiR and green *Ac*NiR showing structural difference of residue Met136.

Met136 is flipped 180° away from T1Cu residue His140 (*Br*^{2D}NiR numbering), allowing a unique hydrogen bond to be formed with a new water (W10). *Br*^{2D}NiR structure and numbering shown in cyan, *Ax*NiR structure and numbering shown in dark blue and *Ac*NiR structure and numbering shown in green.

Figure S4

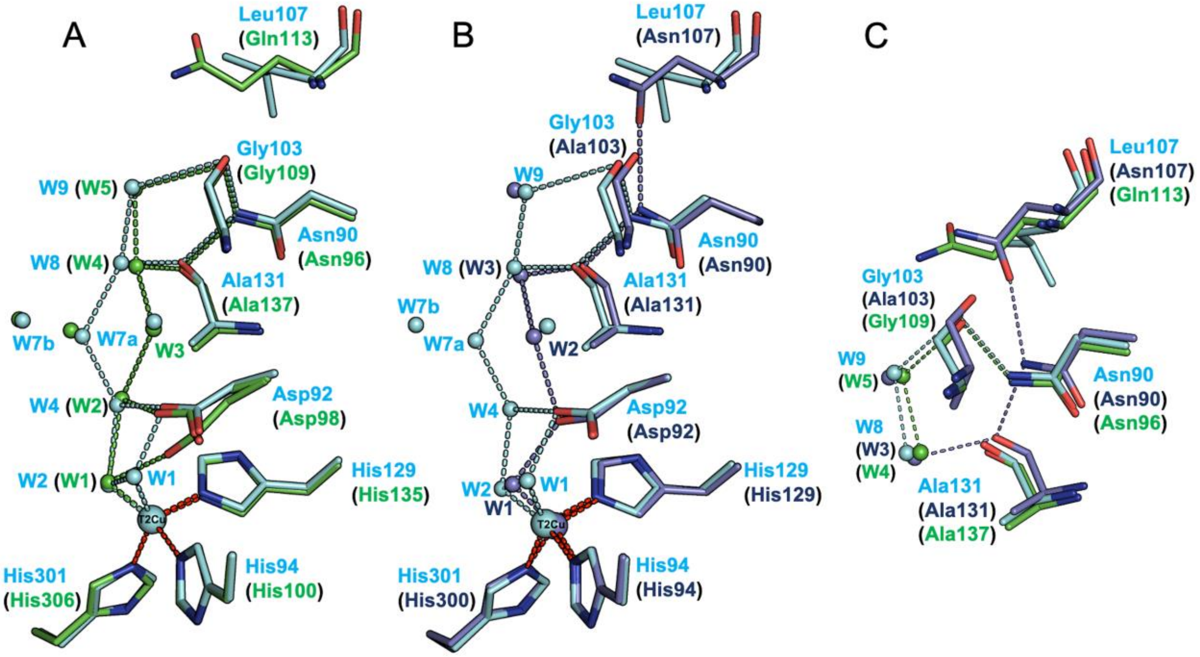

Fig. S4. Comparison of the Asn90 proton channel in XFEL FRIC structures of *Br*^{2D}NiR with atomic resolution structure of blue *Ax*NiR (pdb:10E1) and XFEL FRIC structure of green *Ac*NiR (pdb:6GSQ).

Br^{2D}NiR XFEL FRIC structure and numbering shown in cyan, *Ax*NiR atomic resolution structure and numbering shown in dark blue and XFEL FRIC *Ac*NiR structure and numbering shown in green. (**A**) *Br*^{2D}NiR (cyan) v *Ac*NiR (green) Asn90 channel is very similar in both (Asn-Gly-W-W(Ala)-W-W(Asp)-W(T2Cu)). In *Br*^{2D}NiR half occupancy W7a is favored in the water network compared to W3 in *Ac*NiR due to weak hydrogen bond distance with the aligning water in *Br*^{2D}NiR and W4. (**B**) *Br*^{2D}NiR (cyan) v *Ax*NiR (dark blue) Asn90 channel shows a large difference with less waters making up the pathway in *Ax*NiR (Asn-Asn-Ala-W-W-Asp-W(T2Cu)). (**C**) Superimposed *Br*^{2D}NiR with *Ax*NiR and *Ac*NiR showing the entrance residues to the Asn90 channel. In *Ax*NiR, Asn107 forms the entrance and is able to hydrogen bond to the essential residue Asn90 to constitute the network by hydrogen bonding to Ala131 (*Ax*NiR numbering) and then water in the pathway. In both *Br*^{2D}NiR and *Ac*NiR, the entrance residues Leu107/Gln113 cannot form this hydrogen bond with Asn90, so instead Gly residues in both substitute this by forming a hydrogen bond between its carbonyl oxygen and Asn90 and then bulk solvent.

Figure S5

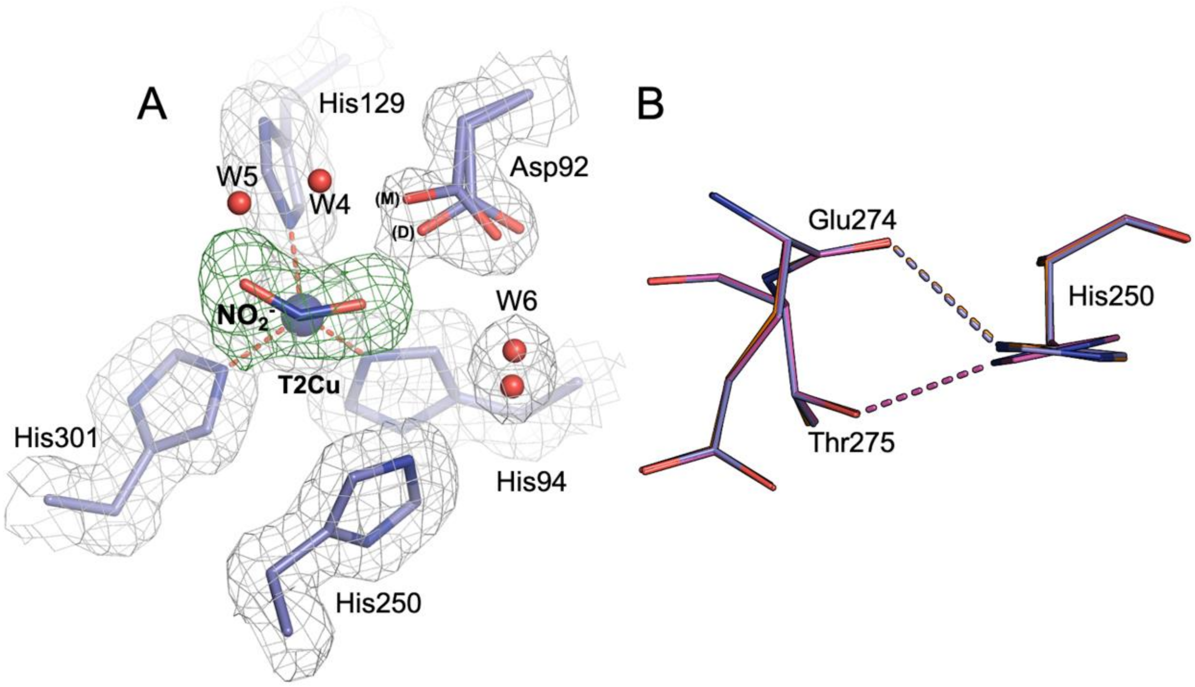

Fig. S5. T2Cu site and His250 (His_{CAT}) rotation in additional low dose nitrite-bound *Br*^{2D}NiR SRX structure at 0.5 MGy.

(**A**) OMIT F_o-F_c electron density map, contoured at 5σ level and colored green around nitrite molecule in 'L-shaped' position. 2F_o-F_c electron density map contoured at 1σ level shows Asp92 in two conformations of proximal position (main and distorted). Coordination to T2Cu is shown as red dashes. (**B**) Confirmation that His_{CAT} rotation in atomic resolution SRX nitrite-bound structure was due to X-ray induced electron transfer after substrate binding: low dose 0.5 MGy SRX structure and damage-free SF-ROX structure are similarly rotated towards to residue Glu274 for suitable hydrogen bond formation, only rotation is seen in the atomic resolution SRX structure which has changed its hydrogen bond to residue Thr275. 0.5 MGy SRX structure is shown by purple sticks and dashes. XFEL FRIC structure is shown by orange sticks and dashes and atomic resolution SRX structure is shown as magenta sticks and dashes.

Table S1. **Data collection and refinement statistics**

	***Br*^{2D}NiR** **as isolated** **SRX 1**	***Br*^{2D}NiR** **as isolated** **SF-ROX 1**	***Br*^{2D}NiR** **nitrite-bound** **SRX 2**	***Br*^{2D}NiR** **nitrite-bound** **SF-ROX 2**	***Br*^{2D}NiR** **NO-bound** **SRX 3**	***Br*^{2D}NiR** **NO-bound** **SF-ROX 3**	***Br*^{2D}NiR** **nitrite-bound** **low dose SRX 4**
**Data collection**							
Space group	$P2_13$	$P2_13$	$P2_13$	$P2_13$	$P2_13$	$P2_13$	$P2_13$
Cell dimensions							
a=b=c (Å)	106.72	107.01	106.79	106.95	107.47	106.87	107.57
Resolution (Å)	47.72-1.10 (1.12-1.10) *	31.0-1.30 (1.35-1.30) *	53.39-1.00 (1.02- 1.00) *	29.8-1.30 (1.35-1.30) *	48.06-1.19 (1.21-1.19) *	31.0-1.30 (1.35-1.30) *	48.15-1.48 (1.50- 1.48) *
R_{merge}	0.069 (0.639)	N/A	0.079 (1.201)	N/A	0.061 (1.358)	N/A	0.094 (1.638)
R_{pim} or R_{split}	0.030 (0.436)	0.115 (0.777)	0.033 (0.731)	0.163 (1.126)	0.025 (0.572)	0.132 (0.340)	0.039 (0.667)
CC(1/2)	0.999 (0.630)	0.981 (0.332)	0.999 (0.388)	0.951 (0.016)	1.00 (0.508)	0.967 (0.472)	0.999 (0.541)
$I / \sigma I$	11.7 (1.2)	5.4 (1.5)	10.8 (0.9)	4.1 (1.5)	16.7 (1.4)	7.1 (3.3)	10.8 (1.1)
Completeness (%)	99.8 (97.0)	100.0 (99.9)	99.9 (99.3)	99.9 (99.5)	99.9 (97.9)	100 (100)	99.9 (98.0)
Redundancy	6.0 (2.7)	121.0 (22.9)	6.3 (3.6)	73.4 (21.1)	6.8 (6.5)	148.1 (94.4)	6.9 (6.9)
Wilson B (Å^2)	7.1	16.7	6.5	16.3	9.7	13.9	14.9
**Refinement**							
Resolution (Å)	30.00-1.10	30.91-1.30	30-1.00	29.68-1.30	30.00-1.19	30.87-1.30	48.15-1.48
No. reflections	162,670	94,957	216,821	99,687	131,757	94,625	65,659
R_{work} / R_{free}	0.125/0.148	0.151/0.206	0.126/0.143	0.156/0.206	0.123/0.126	0.135/0.180	0.147/0.176
No. atoms							
Protein	2739	2850	2776	2870	2814	2780	2930
Ligand/ion	93/2	55/2	112/2	101/2	155/2	154/2	123/2
Water	660	514	771	459	509	516	532
B-factors (Å2)							
Protein	13.2	23.33	11.77	22.95	16.69	18.47	20.83
Ligand/Cu	30.64/10.54	49.18/18.46	27.12/7.77	40.16/18.08	34.26/13.94	38.71/14.97	49.19/16.32
Water	33.02	38.95	39.94	36.84	37.79	34.41	49.19
R.m.s. deviations							
Bond lengths (Å)	0.015**	0.013	0.014**	0.015	0.015**	0.017	0.013
Bond angles (°)	2.26**	1.81	2.25**	1.90	2.25**	2.06	1.95
**PDB ID**	**6ZAR**	**6ZAS**	**6ZAT**	**6ZAU**	**6ZAV**	**6ZAW**	**6ZAX**

Number of crystals used for SRX structures – one,
Number of crystals used for SF-ROX structures – as isolated (40); nitrite-bound (28); NO-bound (55).
* values in parentheses are for highest-resolution shell.
** number is from restrained refinement round

Table S2. T1Cu bonding ligand distances (Å)

	As-isolated SRX 1	Nitrite-bound SRX 2		NO-bound SRX 3	Nitrite-bound low dose SRX 4	As-isolated SF-ROX 1	Nitrite-bound SF-ROX 2	NO-bound SF-ROX 3
T1Cu		T1Cu[b]	T1Cu[a]					
His140$N^{\delta1}$	2.050(10)	2.284(10)	1.950(9)	2.11(2)	1.96	1.97	1.95	2.05
His89$N^{\delta1}$	2.057(9)	2.057(9)	2.037(8)	2.09(1)	2.02	1.96	1.96	2.03
Cys130S^{γ}	2.172(4)	2.136(6)	2.184(4)	2.200(6)	2.16	2.13	2.11	2.20
Met145S^{δ}	2.574(5)	2.341(6)	2.654(4)	2.489(7)	2.54	2.56	2.54	2.47

Table S3. T2Cu bonding ligand distances (Å)

	As-isolated SRX 1	Nitrite-bound SRX 2	NO-bound SRX 3	Nitrite-bound low dose SRX 4	As-isolated SF-ROX 1	Nitrite-bound SF-ROX 2	NO-bound SF-ROX 3
T2Cu							
His301$N^{\varepsilon2}$	2.010(10)	2.006(8)	2.01(1)	2.06	2.06	2.05	1.98
His94$N^{\varepsilon2}$	2.009(10)	1.993(8)	1.99(1)	2.01	2.00	1.98	2.01
His129$N^{\varepsilon2}$	2.083(10)	2.028(7)	1.97(1)	2.07	2.14	2.05	1.96
W2	2.162(3)				2.05		
W1	1.940(3)				1.94		
W3	2.018(3)						
NO_2^{O1} b		2.127(61)		2.33		2.12	
NO_2^{O2}b		1.976(20)		1.92		1.92	
NO_2^{N}b		2.376(23)		1.78		2.02	
NO_2^{O1} a		2.223(52)					
NO_2^{O2}a		2.134(16)					
NO_2^{N}a		2.412(26)					
NO^{N}			2.17(4)				2.47
NO^{O}			2.07(3)				2.12
Wa							2.07

NO_2**b** - 'Top-hat' nitrite**;** NO_2**a** - 'Bent top-hat' nitrite

Table S4. Bond lengths and bond angles in atomic resolution structures, characteristic for protonation.

Distances	As-isolated SRX 1	Nitrite-bound SRX 2	NO-bound SRX 3
Asp_{CAT}			
Main Proximal			
Occupancy	0.67	0.60	1.00
C^{γ}-$O^{\delta1}$ (Å)	1.25(3)	1.25(2)	1.24(3)
C^{γ}-$O^{\delta2}$ (Å)	1.26(3)	1.26(2)	1.28(3)
Distorted proximal			
Occupancy	0.33		
C^{γ}-$O^{\delta1}$ (Å)	1.25(7)		
C^{G}-$O^{\delta2}$ (Å)	1.29(8)		
Gatekeeper			
Occupancy		0.40	
C^{γ}-$O^{\delta1}$ (Å)		1.25(3)	
C^{γ}-$O^{\delta2}$ (Å)		1.26(3)	
His_{CAT}			
$C^{\varepsilon1}$-$N^{\varepsilon2}$-$C^{\delta2}$ (°)	106.4(1.5)	109.4(0.9)	105.0(2.2)
C^{γ} -$N^{\delta1}$-$C^{\varepsilon1}$ (°)	112.5(1.6)	114.0(0.9)	113.6(2.4)
NO_2^-			
Conformation B ('Top-hat')			
Occupancy		0.40	
N-O1 (Å)		1.30(4)	
N-O2 (Å)		1.30(3)	
Conformation A ('Bent top-hat')			
Occupancy		0.60	
N-O1 (Å)		1.25(4)	
N-O2 (Å)		1.28(3)	
NO			
Occupancy			0.73
N-O (Å)			1.20(5)

e.s.d.s values to the last digit in brackets

Table S5. Hydrogen-bond contact distances (Å)

	As-isolated SRX 1	Nitrite-bound SRX 2		NO-bound SRX 3	Nitrite-bound low dose SRX 4	As-isolated SF-ROX 1	Nitrite-bound SF-ROX 2	NO-bound SF-ROX 3
W1 - W2	2.49					2.51		
Asp92 Proximal $O^{\delta1}$								
- W1 (Main)	2.52					2.39		
- W3 (Distorted)	3.13							
- W4 (Main)	2.45	2.51		2.54	2.27	2.81	2.54	2.38
- NO_2^{O2}		1.92 (TH)	3.42 (BTH)		2.36		2.25	
- NO^{O}				2.48				2.56
- Wa								2.16
Asp92 Proximal $O^{\delta2}$								
- W6 (Bridging water)	2.77	3.03		2.86	2.53	2.75	2.83	3.00
His250 $N^{\varepsilon2}$								
- W6 (Bridging water)	3.05	2.94		2.88	3.19	3.10	2.94	2.84
- NO_2^{O2}		3.74 (TH)	3.06 (BTH)		3.39		3.17	
His250 $N^{\delta1}$								
- Glu274 O	2.67	2.72		2.65	2.62	2.64	2.52	2.61
- Thr275 $O^{\gamma1}$	3.09	2.79		2.89	2.95	3.07	2.97	2.80
W_G (with Asp92 Gatekeeper and 'top-hat' nitrite)								
- W6		2.51						
- NO_2^{O2}b		2.73						
- Leu100 O_G		2.69						
W4								
- W2	2.98					2.62		
- W5		2.71						2.08
- NO_2^{O1}		3.05 (TH)	3.12 (BTH)		2.89		3.11	
- NO^{N}				2.65				2.74
W5								
- W3	2.98							
- NO_2^{O}		2.81 (TH)	2.71 (BTH)		2.07			
- NO^{N}				2.02				2.07
His140 - W10	2.75	2.79		2.76	2.90	2.79	2.73	2.92

TH - 'Top-hat' nitrite position**;** **BTH** - 'Bent top-hat' nitrite position**;** NO_2**b** - 'Top-hat' nitrite**;** NO_2**a** - 'Bent top-hat' nitrite

REFERENCES AND NOTES

1. N. Gruber, J. N. Galloway, An Earth-system perspective of the global nitrogen cycle. *Nature* **451**, 293–296 (2008).

2. D. E. Canfield, A. N. Glazer, P. G. Falkowski, The evolution and future of earth's nitrogen cycle. *Science* **330**, 192–196 (2010).

3. G. W. O'Hara, R. M. Daniel, Rhizobial denitrification: A review. *Soil Biol. Biochem.* **17**, 1–9 (1985).

4. W. G. Zumft, Cell biology and molecular basis of denitrification. *Microbiol. Mol. Biol. Rev.* **61**, 533–616 (1997).

5. R. R. Eady, S. S. Hasnain, Denitrification. *Compr. Coord. Chem. II* **8**, 759–786 (2004).

6. G. W. O'Hara, R. M. Daniel, K. W. Steele, P. M. Bonish, Nitrogen losses from soils caused by Rhizobium-dependent denitrification. *Soil Biol. Biochem.* **16**, 429–431 (1984).

7. M. A. J. Delgado, S. Casella, E. J. Bedmar, Denitrification in rhizobia-legume symbiosis, in *Biology of the Nitrogen Cycle*, H. Bothe, S. J. Ferguson, W. E. Newton, Eds. (Elsevier, 2007). pp 83–91

8. M. K. Firestone, R. B. Firestone, J. M. Tiedje, Nitrous oxide from soil denitrification: Factors controlling its biological production. *Science* **208**, 749–751 (1980).

9. A. R. Ravishankara, J. S. Daniel, R. W. Portmann, Nitrous oxide (N2O): The dominant ozone-depleting substance emitted in the 21st century. *Science* **326**, 123–125 (2009).

10. A. Hidalgo-García, M. J. Torres, A. Salas, E. J. Bedmar, L. Girard, M. J. Delgado, Rhizobium etli produces nitrous oxide by coupling the assimilatory and denitrification pathways. *Front. Microbiol.* **10**, 980 (2019).

11. M. Nojiri, Structure and function of copper nitrite reductase, in *Metalloenzymes in Denitrification: Applications and Environmental Impacts*, I. Moura, J. Moura, S. Pauleta, L. Maia, Eds. (The Royal Society of Chemistry, 2016). pp 91–113.

12. K. Kataoka, H. Furusawa, K. Takagi, K. Yamaguchi, S. Suzuki, Functional analysis of conserved aspartate and histidine residues located around the type 2 copper site of copper-containing nitrite reductase. *J. Biochem.* **127**, 345–350 (2000).

13. M. J. Boulanger, M. E. P. Murphy, Directing the mode of nitrite binding to a copper-containing nitrite reductase from Alcaligenes faecalis S-6: Characterization of an active site isoleucine. *Protein Sci.* **12**, 248–256 (2003).

14. M. J. Boulanger, M. Kukimoto, M. Nishiyama, S. Horinouchi, M. E. P. Murphy, Catalytic roles for two water bridged residues (Asp-98 and His-255) in the active site of copper-containing nitrite reductase. *J. Biol. Chem.* **275**, 23957–23964 (2000).

15. A. C. Merkle, N. Lehnert, Binding and activation of nitrite and nitric oxide by copper nitrite reductase and corresponding model complexes. *Dalton Trans.* **41**, 3355–3368 (2012).

16. S. V. Antonyuk, R. W. Strange, G. Sawers, R. R. Eady, S. S. Hasnain, Atomic resolution structures of resting-state, substrate- and product-complexed Cu-nitrite reductase provide insight into catalytic mechanism. *Proc. Natl. Acad. Sci. U.S.A.* **102**, 12041–12046 (2005).

17. M. P. Blakeley, S. S. Hasnain, S. V. Antonyuk, Sub-atomic resolution x-ray crystallography and neutron crystallography: Promise, challenges and potential. *IUCrJ* **2**, 464–474 (2015).

18. Y. Fukuda, K. M. Tse, T. Nakane, T. Nakatsu, M. Suzuki, M. Sugahara, S. Inoue, T. Masuda, F. Yumoto, N. Matsugaki, E. Nango, K. Tono, Y. Joti, T. Kameshima, C. Song, T. Hatsui, M. Yabashi, O. Nureki, M. E. P. Murphy, T. Inoue, S. Iwatae, E. Mizohata, Redox-coupled proton transfer mechanism in nitrite reductase revealed by femtosecond crystallography. *Proc. Natl. Acad. Sci. U.S.A.* **113**, 2928–2933 (2016).

19. F. E. Dodd, S. S. Hasnain, Z. H. L. Abraham, R. R. Eady, B. E. Smith, Structures of a blue-copper nitrite reductase and its substrate-bound complex. *Acta Crystallogr. Sect. D Biol. Crystallogr.* **53**, 406–418 (1997).

20. N. G. H. Leferink, C. Han, S. V. Antonyuk, D. J. Heyes, S. E. J. Rigby, M. A. Hough, R. R. Eady, N. S. Scrutton, S. S. Hasnain, Proton-coupled electron transfer in the catalytic cycle of Alcaligenes xylosoxidans copper-dependent nitrite reductase. *Biochemistry* **50**, 4121–4131 (2011).

21. D. Sasaki, T. F. Watanabe, R. R. Eady, R. C. Garratt, S. V. Antonyuk, S. S. Hasnain, Reverse protein engineering of a novel 4-domain copper nitrite reductase reveals functional regulation by protein-protein interaction. *FEBS J.* doi:10.1111/febs.15324 (2020).

22. S. Ghosh, A. Dey, Y. Sun, C. P. Scholes, E. I. Solomon, Spectroscopic and computational studies of nitrite reductase: Proton induced electron transfer and backbonding contributions to reactivity. *J. Am. Chem. Soc.* **131**, 277–288 (2009).

23. Y. Fukuda, Y. Hirano, K. Kusaka, T. Inoue, T. Tamada, High-resolution neutron crystallography visualizes an OH-bound resting state of a copper-containing nitrite reductase. *Proc. Natl. Acad. Sci. U.S.A.*, 201918125 (2020).

24. T. M. Hedison, M. Shanmugam, D. J. Heyes, R. Edge, N. S. Scrutton, Active intermediates in copper nitrite reductase reactions probed by a cryotrapping-electron paramagnetic resonance approach. *Angew. Chem. Int. Ed.* **59**, 13936–13940 (2020).

25. G. Cioncoloni, I. Roger, P. S. Wheatley, C. Wilson, R. E. Morris, S. Sproules, M. D. Symes, Proton-coupled electron transfer enhances the electrocatalytic reduction of nitrite to NO in a bioinspired copper complex. *ACS Catal.* **8**, 5070–5084 (2018).

26. C. M. Moore, N. K. Szymczak, Nitrite reduction by copper through ligand-mediated proton and electron transfer. *Chem. Sci.* **6**, 3373–3377 (2015).

27. J. K. Bower, A. Y. Sokolov, S. Zhang, Four-coordinate copper halonitrosyl $\{CuNO\}^{10}$ complexes. *Angew. Chem. Int. Ed.* **58**, 10225–10229 (2019).

28. G. M. Sheldrick, A short history of SHELX. *Acta Crystallogr. Sect. A Found. Crystallogr.* **64**, 112–122 (2008).

29. T. P. Halsted, K. Yamashita, C. C. Gopalasingam, R. T. Shenoy, K. Hirata, H. Ago, G. Ueno, M. P. Blakeley, R. R. Eady, S. V. Antonyuk, M. Yamamoto, S. S. Hasnain, Catalytically important damage-free structures of a copper nitrite reductase obtained by femtosecond X-ray laser and room-temperature neutron crystallography. *IUCrJ* **6**, 761–772 (2019).

30. M. Malinska, M. Dauter, M. Kowiel, M. Jaskolski, Z. Dauter, Protonation and geometry of histidine rings. *Acta Crystallogr. Sect. D Biol. Crystallogr.* **71**, 1444–1454 (2015).

31. D. Liebschner, M. Dauter, A. Brzuszkiewicz, Z. Dauter, On the reproducibility of protein crystal structures: Five atomic resolution structures of trypsin. *Acta Crystallogr. Sect. D Biol. Crystallogr.* **69**, 1447–1462 (2013).

32. F. H. Allen, The Cambridge Structural Database: A quarter of a million crystal structures and rising. *Acta Crystallogr. Sect. B Struct. Sci.* **58**, 380–388 (2002).

33. K. Hirata, K. Shinzawa-Itoh, N. Yano, S. Takemura, K. Kato, M. Hatanaka, K. Muramoto, T. Kawahara, T. Tsukihara, E. Yamashita, K. Tono, G. Ueno, T. Hikima, H. Murakami, Y. Inubushi, M. Yabashi, T. Ishikawa, M. Yamamoto, T. Ogura, H. Sugimoto, J. R. Shen, S. Yoshikawa, H. Ago, Determination of damage-free crystal structure of an x-ray-sensitive protein using an XFEL. *Nat. Methods* **11**, 734–736 (2014).

34. T. P. Halsted, K. Yamashita, K. Hirata, H. Ago, G. Ueno, T. Tosha, R. R. Eady, S. V. Antonyuk, M. Yamamoto, S. S. Hasnain, An unprecedented dioxygen species revealed by serial femtosecond rotation crystallography in copper nitrite reductase. *IUCrJ* **5**, 22–31 (2018).

35. R. W. Strange, L. M. Murphy, F. E. Dodd, Z. H. L. Abraham, R. R. Eady, B. E. Smith, S. S. Hasnain, Structural and kinetic evidence for an ordered mechanism of copper nitrite reductase. *J. Mol. Biol.* **287**, 1001–1009 (1999).

36. S. J. Fisher, M. P. Blakeley, M. Cianci, S. McSweeney, J. R. Helliwell, Protonation-state determination in proteins using high-resolution x-ray crystallography: Effects of resolution and completeness. *Acta Crystallogr. Sect. D Biol. Crystallogr.* **68**, 800–809 (2012).

37. S. Horrell, S. V. Antonyuk, R. R. Eady, S. S. Hasnain, M. A. Hough, R. W. Strange, Serial crystallography captures enzyme catalysis in copper nitrite reductase at atomic resolution from one crystal. *IUCrJ* **3**, 271–281 (2016).

38. S. Horrell, D. Kekilli, K. Sen, R. L. Owen, F. S. N. Dworkowski, S. V. Antonyuk, T. W. Keal, C. W. Yong, R. R. Eady, S. S. Hasnain, R. W. Strange, M. A. Hough, Enzyme catalysis captured using multiple structures from one crystal at varying temperatures. *IUCrJ* **5**, 283–292 (2018).

39. Y. Fukuda, K. M. Tse, M. Lintuluoto, Y. Fukunishi, E. Mizohata, H. Matsumura, H. Takami, M. Nojiri, T. Inoue, Structural insights into the function of a thermostable copper-containing nitrite reductase. *J. Biochem.* **155**, 123–135 (2014).

40. E. I. Tocheva, F. I. Rosell, A. G. Mauk, M. E. P. Murphy, Side-on copper-nitrosyl coordination by nitrite reductase. *Science* **304**, 867–870 (2004).

41. G. Periyasamy, M. Sundararajan, I. H. Hillier, N. A. Burton, J. J. W. McDouall, The binding of nitric oxide at the Cu(I) site of copper nitrite reductase and of inorganic models: DFT calculations of the energetics and EPR parameters of side-on and end-on structures. *Phys. Chem. Chem. Phys.* **9**, 2498–2506 (2007).

42. M. Sundararajan, I. H. Hillier, N. A. Burton, Mechanism of nitrite reduction at T2Cu centers: Electronic structure calculations of catalysis by copper nitrite reductase and by synthetic model compounds. *J. Phys. Chem. B* **111**, 5511–5517 (2007).

43. S. A. De Marothy, M. R. A. Blomberg, P. E. M. Siegbahn, Elucidating the mechanism for the reduction of nitrite by copper nitrite reductase—A contribution from quantum chemical studies. *J. Comput. Chem.* **28**, 528–539 (2007).

44. Y. Li, M. Hodak, J. Bernholc, Enzymatic mechanism of copper-containing nitrite reductase. *Biochemistry* **54**, 1233–1242 (2015).

45. Y. Fukuda, K. M. Tse, M. Suzuki, K. Diederichs, K. Hirata, T. Nakane, M. Sugahara, E. Nango, K. Tono, Y. Joti, T. Kameshima, C. Song, T. Hatsui, M. Yabashi, O. Nureki, H. Matsumura, T. Inoue,

S. Iwata, E. Mizohata, Redox-coupled structural changes in nitrite reductase revealed by serial femtosecond and microfocus crystallography. *J. Biochem.* **159**, 527–538 (2016).

46. N. G. H. Leferink, S. V. Antonyuk, J. A. Houwman, N. S. Scrutton, R. R. Eady, S. S. Hasnain, Impact of residues remote from the catalytic centre on enzyme catalysis of copper nitrite reductase. *Nat. Commun.* **5**, 4395 (2014).

47. S. Brenner, D. J. Heyes, S. Hay, M. A. Hough, R. R. Eady, S. S. Hasnain, N. S. Scrutton, Demonstration of proton-coupled electron transfer in the copper-containing nitrite reductases. *J. Biol. Chem.* **284**, 25973–25983 (2009).

48. M. A. Hough, J. Conradie, R. W. Strange, S. V. Antonyuk, R. R. Eady, A. Ghosh, S. S. Hasnain, Nature of the copper-nitrosyl intermediates of copper nitrite reductases during catalysis. *Chem. Sci*. 10.1039/d0sc04797j (2020).

49. G. Winter, D. G. Waterman, J. M. Parkhurst, A. S. Brewster, R. J. Gildea, M. Gerstel, L. Fuentes-Montero, M. Vollmar, T. Michels-Clark, I. D. Young, N. K. Sauter, G. Evans, DIALS: Implementation and evaluation of a new integration package. *Acta Crystallogr. Sect. D Struct. Biol.* **74**, 85–97 (2018).

50. G. Winter, Xia2: An expert system for macromolecular crystallography data reduction. *J. Appl. Crystallogr.* **43**, 186–190 (2010).

51. W. Kabsch, XDS. *Acta Crystallogr. D Biol. Crystallogr.* **66**, 125–32 (2010).

52. K. Yamashita, K. Hirata, M. Yamamoto, KAMO: Towards automated data processing for microcrystals. *Acta Crystallogr. Sect. D Struct. Biol.* **74**, 441–449 (2018).

53. G. N. Murshudov, P. Skubák, A. A. Lebedev, N. S. Pannu, R. A. Steiner, R. A. Nicholls, M. D. Winn, F. Long, A. A. Vagin, REFMAC5 for the refinement of macromolecular crystal structures. *Acta Crystallogr. Sect. D Biol. Crystallogr.* **67**, 355–367 (2011).

54. M. D. Winn, C. C. Ballard, K. D. Cowtan, E. J. Dodson, P. Emsley, P. R. Evans, R. M. Keegan, E. B. Krissinel, A. G. W. Leslie, A. McCoy, S. J. McNicholas, G. N. Murshudov, N. S. Pannu, E. A.

Potterton, H. R. Powell, R. J. Read, A. Vagin, K. S. Wilson, Overview of the CCP4 suite and current developments. *Acta Crystallogr. Sect. D Biol. Crystallogr.* **67**, 235–242 (2011).

55. P. Emsley, K. Cowtan, Coot: Model-building tools for molecular graphics. *Acta Crystallogr. Sect. D Biol. Crystallogr.* **60**, 2126–2132 (2004).

56. T. A. White, V. Mariani, W. Brehm, O. Yefanov, A. Barty, K. R. Beyerlein, F. Chervinskii, L. Galli, C. Gati, T. Nakane, A. Tolstikova, K. Yamashita, C. H. Yoon, K. Diederichs, H. N. Chapman, Recent developments in CrystFEL. *J. Appl. Crystallogr.* **49**, 680–689 (2016).

57. A. J. M. Duisenberg, Indexing in single-crystal diffractometry with an obstinate list of reflections. *J. Appl. Crystallogr.* **25**, 92–96 (1992).

58. H. R. Powell, O. Johnson, A. G. W. Leslie, Autoindexing diffraction images with iMosflm. *Acta Crystallogr. Sect. D Biol. Crystallogr.* **69**, 1195–1203 (2013).

59. O. B. Zeldin, M. Gerstel, E. F. Garman, RADDOSE-3D: Time- and space-resolved modelling of dose in macromolecular crystallography. *J. Appl. Crystallogr.* **46**, 1225–1230 (2013).
